# Supplementary material for: The active sites of Cu–ZnO catalysts for water gas shift and CO hydrogenation reactions
Source: Nat Commun. 2021 Jul 15;12:4331. doi: 10.1038/s41467-021-24621-8 (PMC8282834; doi:10.1038/s41467-021-24621-8)
Supplement: Supplementary file 1 — Supplementary Information [file 41467_2021_24621_MOESM1_ESM.pdf]

Supplementary Information

The active sites of Cu-ZnO catalysts for water gas shift and CO hydrogenation reactions

Zhang *et al.*

Supplementary Methods

Powder X-ray diffraction (XRD) patterns were performed on a Philips X'Pert PROS diffractometer using a nickel-filtered Cu Kα (wavelength: 0.15418 nm) radiation source with an operation voltage of 40 kV and an operation current of 50 mA. X-ray photoelectron spectroscopy (XPS) were conducted on an ESCALAB 250 high performance electron spectrometer using monochromatized Al Kα (hν = 1486.7 eV) as the excitation source. The likely charging of samples was corrected by setting the binding energy of the adventitious carbon (C1s) to 284.8 eV. The specific BET surface areas were measured using Micromeritics Tristar II 3020M. Prior to the measurement, the sample was degassed at 423 K for 5 h in a N_2 atmosphere. The contents of ZnO were measured on an Optima 7300 DV inductively coupled plasma atomic emission spectrometer (ICP-AES). Scanning electron microscope (SEM) images were recorded on a JEOL JSM-6700 field emission scanning electron microscope. Transmission electron microscopy (TEM) and high-resolution transmission electron (HRTEM) images were recorded on a JEM-2100F high resolution transmission electron microscope.

H_2 temperature-programmed reduction (H_2-TPR) and CO temperature-programmed reduction (CO-TPR) experiments were conducted on a Micromeritics Autochem 2920 apparatus equipped with a TCD detector connected with an online HIDEN QIC-20 gas phase mass spectra. In H_2-TPR experiments, 20 mg of as-synthesized Cu_2O or ZnO/Cu_2O catalysts was placed in a U-shaped quartz microreactor and then heated at a rate of 5 K min^{-1} in 5% H_2/Ar with a flow rate of 30 mL min^{-1}. The H_2 consumption signal was recorded by TCD detector. In CO-TPR experiments, 30 mg of as-synthesized Cu_2O and ZnO/Cu_2O catalysts was placed in a U-shaped quartz microreactor and then heated at a rate of 5 K min^{-1} under 5% CO/Ar with a flow rate of 30 mL min^{-1}. The generated CO_2 was measured by an online HIDEN QIC-20 gas phase mass spectra.

Experiments of temperature-programmed reaction spectra (TPRS) were also conducted on a Micromeritics ChemiSorb 2750 connected with an online HIDEN QIC-20 gas phase mass spectra to detect the compositions of the effluent gas. In CO+H_2O-TPRS experiments with low reactant contents, 100 mg of as-synthesized Cu_2O NCs was placed in a quartz microreactor to prepare corresponding Cu NCs by the in-situ reduction in 5% CO/Ar atmosphere. The reduction conditions are same to the descriptions of catalyst preparations. Then, the sample was heated to 548 K at a rate of 5 K min^{-1} in a gas stream consisting of 0.432% CO and 278 K water vapor (water vapor pressure: 0.8726 kPa) balanced with Ar at a flow rate of 30 mL min^{-1}. The P_{CO}:P_{H2O} ratio is 1:2 in the stream. In CO+H_2O-TPRS experiments under working conditions, the Cu or ZnO/Cu catalysts were acquired by in situ reduction of 100 mg of as-synthesized Cu_2O or ZnO/Cu_2O catalysts described in catalyst preparations and then heated to 548 K at a rate of 5 K min^{-1} in a gas stream consisting of 5% CO and 319 K water vapor (water vapor pressure: 10.094 kPa) balanced with Ar at a flow rate of 30 mL min^{-1}. The P_{CO}:P_{H2O} ratio is also 1:2 in the stream.

XPS experiments without exposure to air were conducted on an ESCALAB 250 high performance electron spectrometer equipped with a high-pressure reactor using monochromatized Al Kα (hν = 1486.7 eV) as the excitation source. As-synthesized Cu_2O or ZnO/Cu_2O catalysts were first reduced under desired conditions at ambient pressure in the high-pressure reactor to prepare corresponding Cu or ZnO/Cu catalysts. The reduction conditions are same to the descriptions of catalyst preparations. The acquired sample was cooled to room temperature naturally and then pumped and transferred to the analysis chamber for the XPS measurements without exposures to air.

Near ambient pressure XPS (NAP-XPS) experiments were conducted on a commercialized system equipped with a SPECS PHOIBOS 150 NAP hemispherical analyzer and monochromatic SPECS μ-FOCUS 600 NAP X-ray source: Al Kα line (hν =

1486.6 eV). This system allows NAP-XPS sample investigations in the pressure range for gasses up to 25 mbar with sample temperatures up to 1400 K by laser heating. In $CO+H_2O$ experiments, the sample was first pretreated under 1 mbar CO atmosphere at desired temperature for 1 h to acquire fresh Cu or ZnO/Cu catalysts. After cooling to room temperature, the XPS spectra of fresh surface were recorded. Next, the mixture consisting of 0.33 mbar CO and 0.67 mbar H_2O was introduced via a leak valve and kept at different temperatures (323 K, 423K and 523 K) for 1 h. The corresponding XPS spectra were recorded under in situ atmosphere at different reaction temperatures. In the H_2O activation experiments, the acquired Cu or ZnO/Cu catalysts were first treated under 1 mbar H_2O atmosphere at different temperatures (323 K, 423K and 523 K) for 1 h. The corresponding XPS spectra were recorded. Subsequently, the H_2O was extracted after sample subjected to water activation at 523 K and next 1 mbar CO was introduced at 523 K for 1 h. The XPS spectra were recorded under in situ atmosphere to investigate the reactivity of water-activated sample toward CO.

In-situ Diffuse reflectance infrared Fourier transformed spectroscopy (DRIFTS) experiments were performed on a Nicolet 6700 FT-IR spectrometer equipped with an in situ low-temperature and high-vacuum DRIFTS reaction cell (Harrick Scientific Products, INC) using an MCT/A detector in the series mode with 256 scans and at a resolution of 4 cm^{-1}. In the CO adsorption experiments, 100 mg of as-synthesized Cu_2O or ZnO/Cu_2O catalysts were loaded on the sample stage of the reaction cell and reduced under 5% CO/Ar (flow rate: 30 mL min^{-1}) at desired temperature described in catalyst preparations for 2 h to acquired corresponding Cu or ZnO/Cu catalysts. Then the reaction cell was pumped to a pressure of 0.01 Pa and subsequently cooled to 123 K. The spectra were taken as the background spectra. Next high-pure CO was admitted via a leak value into the reaction cell to reach a pressure of 400 Pa and the DRIFTS spectra were recorded after CO adsorption reached the steady state. In the $CO+H_2$ experiments under high

pressure, Cu or ZnO/Cu catalysts were first acquired by in situ reduction of corresponding Cu_2O or ZnO/Cu_2O catalysts in 5% H_2/Ar at 523 K for 2 h. Then the reaction cell was pressured to 2 MPa in high-pure Ar and heated to 523 K. The spectra were taken as the background spectra. Subsequently, the mixture consisting of 33.3% CO and 66.7% H_2 was introduced. The DRIFTS spectra under CO+H_2 atmosphere at 523 K for different times were recorded.

The transmission IR (FT-IR) spectra of CO adsorption was conducted on a CRCP-7070-C type FT-IR spectrometer equipped with an in situ FT-IR reaction cell (Xianquan Industrial and Trading Co., Ltd.) using an MCT/A detector in the series mode with 256 scans and at a resolution of 4 cm^{-1}. Prior to CO adsorption measurements, as-synthesized Cu_2O or ZnO/Cu_2O catalysts were first reduced in 5%H_2/Ar at 523 K for 2 h to prepare corresponding Cu or ZnO/Cu catalysts. The acquired sample was pretreated under CO+H_2 atmosphere (Total pressure: 2 MPa, CO:H_2=1:2) at 523 K for 5 h and then cooled to room temperature. Subsequently, the pressure was drained off and pumped to a pressure of 0.001 Pa, and the sample was cooled to 123 K. The spectra were taken as the background spectra. Next high-pure CO was admitted via a leak value into the reaction cell to reach a pressure of 1000 Pa and the DRIFTS spectra were recorded after CO adsorption reached the steady state.

The catalytic activities of various Cu or ZnO/Cu catalysts in low-temperature water-gas-shift (WGS) reaction were evaluated in a fixed-bed flow reactor. The reactant gas consisted of 5% CO and water vapor at 319 K (water vapor pressure: 10.094 kPa) balanced with Ar to yield the P_{CO}:P_{H2O} ratio of 1:2. 50 mg of Cu_2O or ZnO/Cu_2O diluted with 50 mg of Al_2O_3 were first reduced under 5% CO/Ar with a flow rate of 30 mL min^{-1} at desired temperature described in catalyst preparations for 2 h to acquire corresponding Cu or ZnO/Cu catalysts. The resulting sample was heated to the desired reaction temperatures at a rate of 2 K min^{-1} and kept for different times. The compositions of the

effluent gas were analyzed with an on-line gas chromatography (FULI 9790II) equipped with a thermal conductivity detector and a 5A zeolite column. The catalytic activity was calculated by the change in CO concentration of the inlet and outlet gases. The catalytic activity of commercial $Cu/ZnO/Al_2O_3$ catalyst was evaluated using the same procedure as above after carefully pre-activated in H_2.

The catalytic activities of various Cu or ZnO/Cu catalysts in CO hydrogenation to methanol reaction were evaluated in a high pressure fixed-bed flow reactor. As-synthesized Cu_2O or ZnO/Cu_2O catalysts were first reduced in $5\%H_2/Ar$ with a flow rate of 30 mL min^{-1} at 523 K for 2 h to prepare corresponding Cu or ZnO/Cu catalysts. After cooling to room temperature naturally, the resulting sample was heated to 523 K under $CO+H_2$ mixture ($CO:H_2=1:2$) with a pressure of 2 MPa at a heating rate of 2 K min^{-1}. The reaction pressure of 2 MPa was used in order to match with the maximum pressure of in situ characterizations. The gaseous hourly space velocity (GHSV) is 3600 mL g^{-1} h^{-1} for all catalysts. The compositions of the effluent gas at reaction time of 5 h were analyzed with an on-line gas chromatography (Ruimin GC2060, Shanghai) equipped with a thermal conductivity detector (TCD) and a flame ionization detector (FID). A TDX-01 packed column was connected to the TCD for the separation and analyses of CO, CH_4 and CO_2. A RT-Q-BOND-PLOT capillary column was connected to the FID for the separation and analyses of organic oxygenated compounds and hydrocarbons. The catalytic activity was calculated by the change in CO concentration of the inlet and outlet gases (Eq. 1). The catalytic selectivities of various products were calculated based on the ratio of the single product compared to that of the overall products (Eq. 2-5) (carbon balance was taken into consideration).

$$\text{Conv.} = (n_{CO,in} - n_{CO,out}) / n_{CO,in} \quad \text{(Eq. 1)}$$

$$\text{Selectivity}_{MeOH} = n_{MeOH} / (n_{MeOH} + n_{CO2} + n_{CH4} + x n_{C2+}) \quad \text{(Eq. 2)}$$

$$\text{Selectivity}_{CO2} = n_{CO2} / (n_{MeOH} + n_{CO2} + n_{CH4} + x n_{C2+}) \quad \text{(Eq. 3)}$$

$Selectivity_{CH4} = n_{CH4} / (n_{MeOH} + n_{CO2} + n_{CH4} + xn_{C2+})$ (Eq. 4)

$Selectivity_{C2+} = xn_{C2+} / (n_{MeOH} + n_{CO2} + n_{CH4} + xn_{C2+})$ (Eq. 5)

In which ‘n’ represent the substance content and ‘*x*’ is carbon chain length of C^{2+} components.

The DFT calculations were carried out using the Vienna ab-initio simulation package(VASP) [1,2] using the projector-augment wave (PAW) method [3]. The nonlocal exchange correlation energy was performed via the generalized gradient approximation (GGA) and PBE functional [4,5]. A plane wave basis set with a 400 eV cut off kinetic energy and a 5×5×1 Monkhorst-Pack k-point grid to sample the surface Brillouin zone were performed to give converged results. The energies were converged to within 10-5 eV/atom, and the forces were converged to within 0.02 eV/Å.

The Cu(111) and Cu(100) surface were modeled using a three-layer slab with (5 × 6) surface unit cell. A (1 × 4)-11-layer Cu(211) slab and a (1 × 2)-22-layer Cu(611) slab with 1/4 Cu substituted by Zn on the step edge, denoted as Cu(211)-Zn and Cu(611)-Zn, were used to simulate CuZn alloy [6,7]. One-layer graphite-like (1 × 4) ZnO(0001) ribbon, with an in-plane lattice of 3.30 Å, on three-layer (5 × 6) Cu(111) and three-layer (5 × 6) Cu(100) slab were adopted to simulate Cu/ZnO interface according to previous results [8-12]. A vacuum region of 15 Å between any two repeated slabs was found to be sufficient to avoid interactions between repeated slabs along the z-direction. The top two layers of Cu/ZnO, top six layers of Cu(211)-Zn, top twelve layers of Cu(611)-Zn, and the adsorbed species were fully relaxed, and the remaining layers were fixed in their bulk truncated positions.

The adsorption energies (E_{ads}) were calculated at their most stable structures, relative to the clean surfaces and the isolated atoms

$E_{ads} = E_{ads/sub} – E_{ads} - E_{sub}$

In which $E_{ads/sub}$, E_{ads}, and E_{sub} are the energy of the optimized adsorption system of adsorbate and substrate, adsorbate in the gas phase, and the clean substrate, respectively.

All transition states (TSs) were located by the force reversed method [13] and climbing-image nudged elastic band method (CINEB) [14,15]. We also performed DFT+U calculations for the correction of the on-site Coulomb repulsion of 3d electrons of Zn atoms. We have tested the two commonly used U-J values of 4.7 eV [16].

The transition states were verified by vibrational analysis showing a single imaginary mode. Zero-point energies and entropy corrections were neglected. The activation energies (E_a) and reaction energies of elementary reactions (E_r) were taken with respect to isolated reactants/products. The negative values of E_r represent exothermic processes, and positive values of E_r represent endothermic processes.

Supplementary Figures and Tables

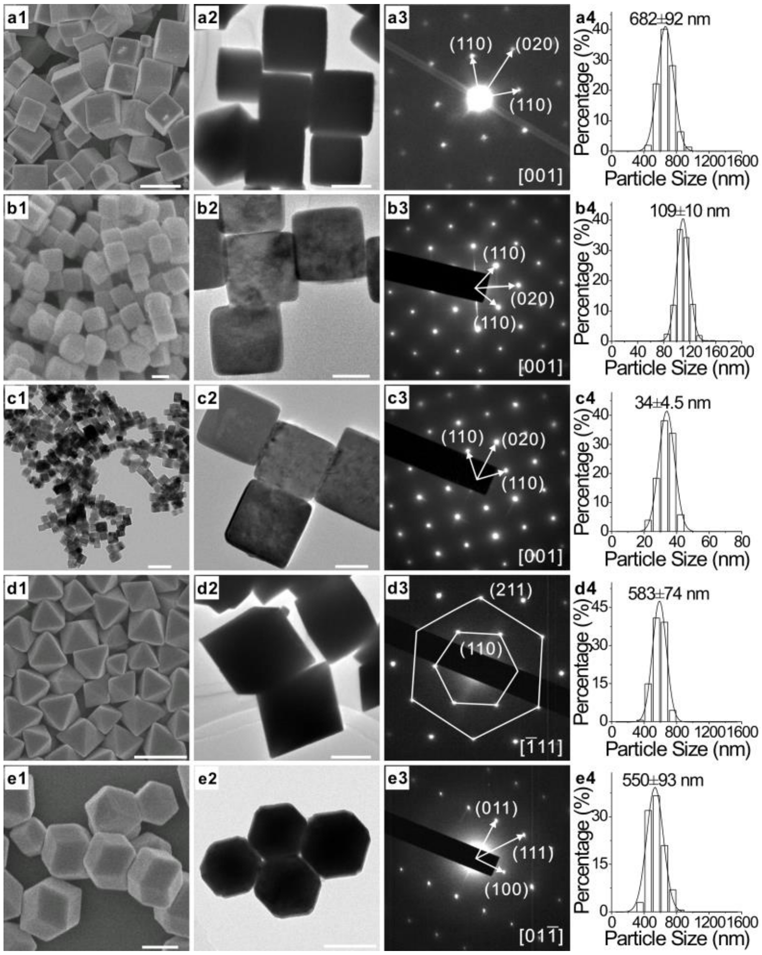

Supplementary Figure 1. The scale bars of (**a1**), (**d1**) correspond to 1 μm, those of (**e1**), (**a2**), (**d2**) and (**e2**) correspond to 500 nm, those of (**b1**) and (**c1**) correspond to 100 nm, that of (**b2**) corresponds to 50 nm, and that of (**c2**) corresponds to 20 nm. SEM, TEM, electron diffraction (ED) patterns, and size distributions of (**a1-a4**) c-Cu_2O-682, (**b1-b4**) c-Cu_2O-109, (**d1-d4**) o-Cu_2O, and (**e1-e4**) d-Cu_2O NCs; TEM, electron diffraction pattern, and size distribution of (**c1-c4**) c-Cu_2O-34 NCs. These results demonstrate that the sizes of all Cu_2O NCs are uniform, and c-Cu_2O-682, c-Cu_2O-109, and c-Cu_2O-34 NCs selectively expose with {100} crystal planes, o-Cu_2O NCs selectively expose with {111} crystal planes, and d-Cu_2O NCs selectively expose with {110} crystal planes, respectively.

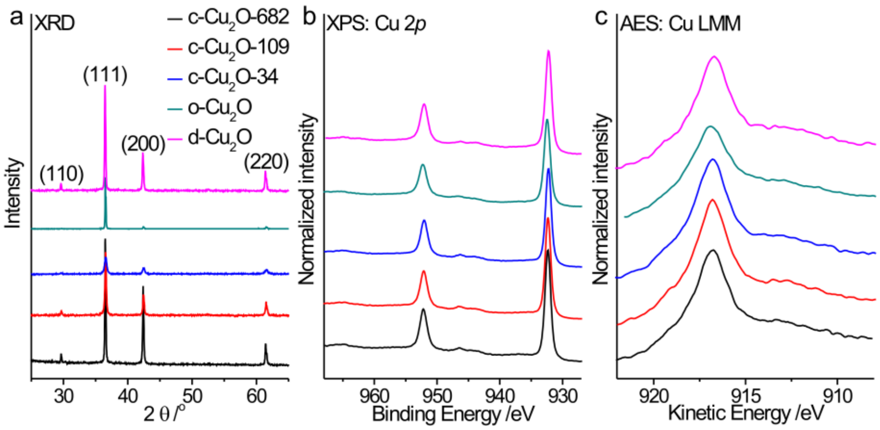

Supplementary Figure 2. (**a**) XRD, (**b**) Cu 2*p* XPS, and (**c**) Cu LMM AES spectra of c-Cu_2O-682, c-Cu_2O-109, c-Cu_2O-34, o-Cu_2O, and d-Cu_2O NCs. The XRD, Cu 2*p* XPS, Cu LMM AES results confirm that both the bulk and surface of all NCs are Cu_2O.

Supplementary Table 1. Specific BET surface areas (m^2 g) of various Cu_2O NCs and the ZnO loadings (wt. %) of various ZnO/Cu catalysts determined by ICP-AES.

Catalysts	BET surface areas (m^2 g)	ZnO loadings (wt. %)	Catalysts	BET surface areas (m^2 g)	ZnO loadings (wt. %)
c-Cu_2O-682	0.96		1%ZnO/c-Cu-34		0.97
c-Cu-682	1.51		3%ZnO/c-Cu-34		2.51
0.5%ZnO/c-Cu-682		0.43	5%ZnO/c-Cu-34		3.90
1%ZnO/c-Cu-682		0.99	7%ZnO/c-Cu-34		5.73
2%ZnO/c-Cu-682		1.90	9%ZnO/c-Cu-34		7.80
5%ZnO/c-Cu-682		3.89	11%ZnO/c-Cu-34		9.63
c-Cu_2O-109	10.38		o-Cu_2O	2.50	
c-Cu-109	2.50		o-Cu	4.09	
1%ZnO/c-Cu-109		0.92	1%ZnO/o-Cu		0.98
2%ZnO/c-Cu-109		1.83	5%ZnO/o-Cu		3.91
3%ZnO/c-Cu-109		2.53	d-Cu_2O	1.98	
5%ZnO/c-Cu-109		3.97	d-Cu	3.33	
7%ZnO/c-Cu-109		5.71	1%ZnO/d-Cu		0.98
9%ZnO/c-Cu-109		7.84	5%ZnO/d-Cu		3.92
c-Cu_2O-34	24.01		Cu/ZnO/Al_2O_3[a]		33.7 (46.9)[c]
c-Cu-34	6.59		Cu/ZnO/Al_2O_3[b]		28.6 (58.2)[c]

[a] Commercial Cu/ZnO/Al_2O_3 catalyst for water-gas shift reaction, purchased from Alfa Aesar Chemical Co. Ltd.;

[b] Commercial $Cu/ZnO/Al_2O_3$ catalyst for CO hydrogenation to methanol reaction, purchased from Alfa Aesar Chemical Co. Ltd.;

[c] Cu loadings (wt. %) determined by ICP-AES.

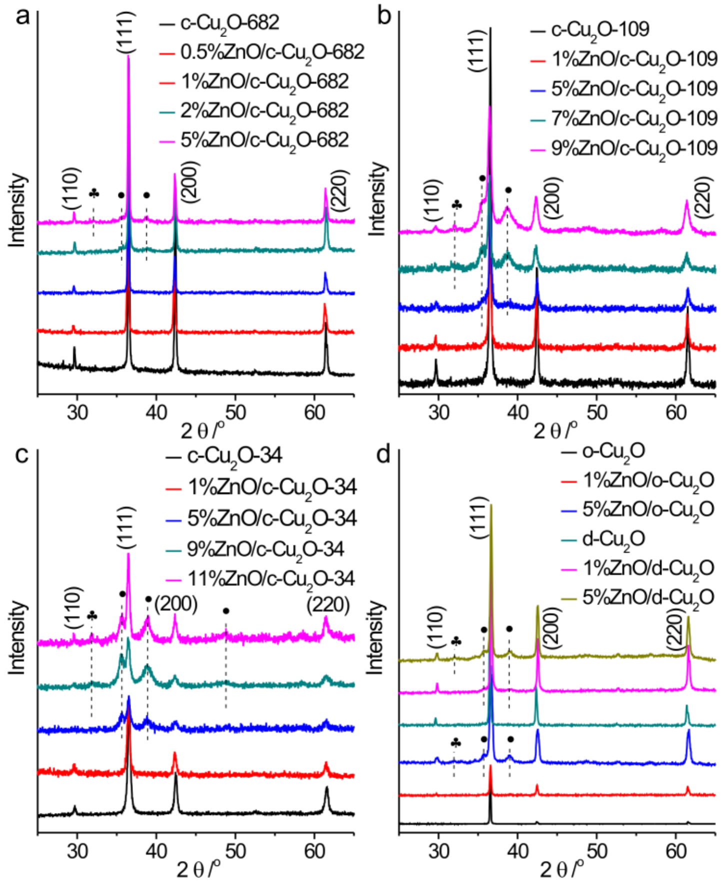

Supplementary Figure 3. XRD patterns of (**a**) c-Cu_2O-682 NCs and various ZnO/c-Cu_2O-682 catalysts, (**b**) c-Cu_2O-109 NCs and various ZnO/c-Cu_2O-109 catalysts, (**c**) c-Cu_2O-34 NCs and various ZnO/c-Cu_2O-34 catalysts, and (**d**) o&d-Cu_2O NCs and various ZnO/o&d-Cu_2O catalysts (♣: ZnO; ●: CuO). The crystal phases of all catalysts are dominant by Cu_2O, while CuO and ZnO crystal phases also appear in ZnO/Cu_2O catalysts with high ZnO loadings supported. The generations of CuO and ZnO crystal phases likely arise from the oxidation of Cu_2O by oxynitride formed by the decomposition of $Zn(NO_3)_2$ precursor and the high ZnO loading leading to the increase of ZnO particle size, respectively.

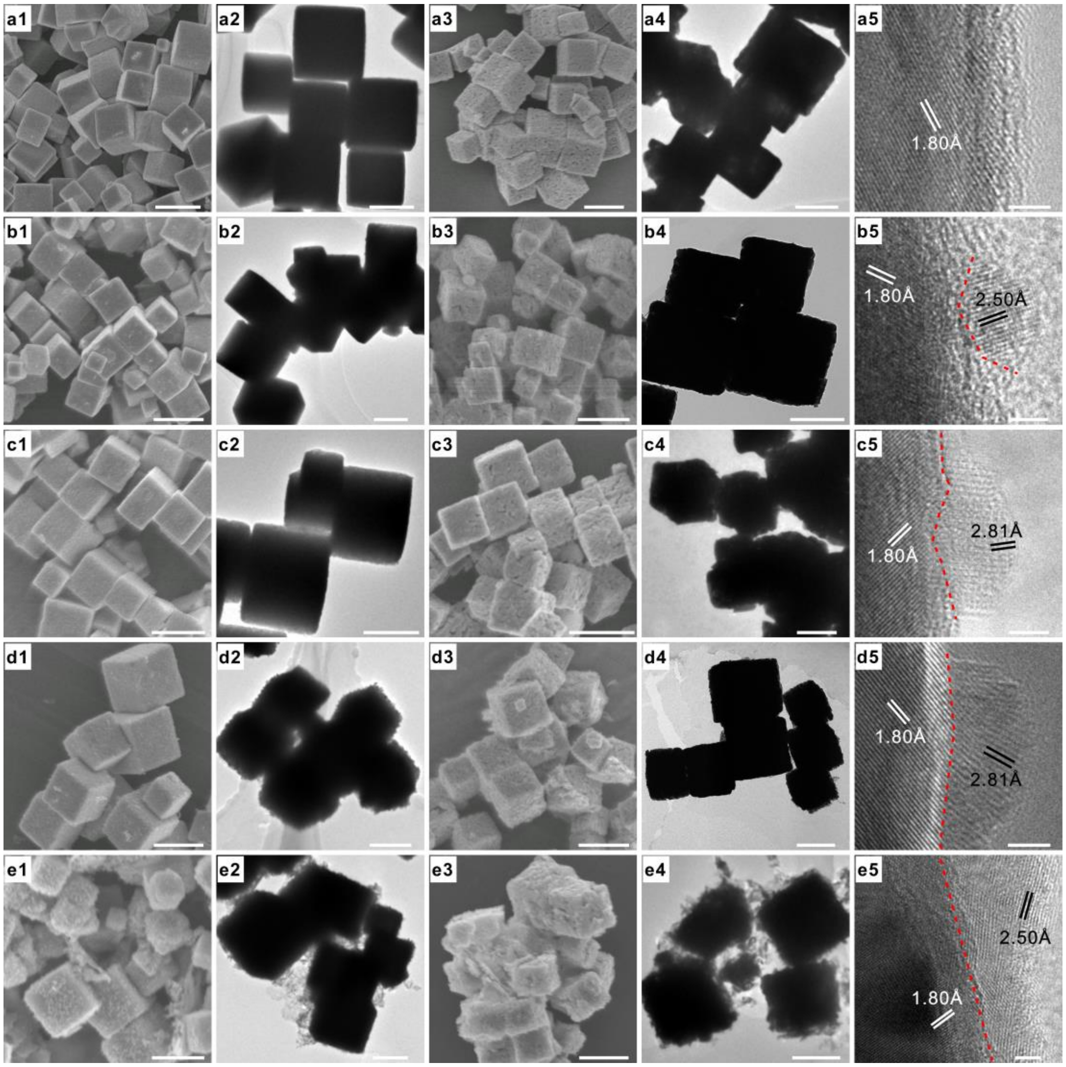

Supplementary Figure 4. The scale bars of (**a1-e1**) and (**a3-e3**) correspond to 1 μm, those of (**a2-e2**) and (**a4-e4**) correspond to 500 nm, and those of (**a5-e5**) correspond to 2 nm. SEM and TEM images of (**a1, a2**) c-Cu_2O-682, (**b1, b2**) 0.5%ZnO/c-Cu_2O-682, (**c1, c2**) 1%ZnO/c-Cu_2O-682, (**d1, d2**) 2%ZnO/c-Cu_2O-682, and (**e1, e2**) 5%ZnO/c-Cu_2O-682 catalysts; SEM, TEM, and HRTEM images of (**a3-a5**) c-Cu-682, (**b3-b5**) 0.5%ZnO/c-Cu-682, (**c3-c5**) 1%ZnO/c-Cu-682, (**d3-d5**) 2%ZnO/c-Cu-682, and (**e3-e5**) 5%ZnO/c-Cu-682 catalysts. Lattice fringes of 1.80, 2.50, and 2.81 Å respectively correspond to the spacing of Cu{200} (JCPDS card NO. 89-2838), and hexagonal ZnO{101} and ZnO{100} (JCPDS card NO 89-1397) crystal planes. For various ZnO/c-Cu_2O-682 catalysts, the original cubic morphology keeps almost unchanged after

ZnO supported and their surfaces attach with ad-particles. As well, the corresponding c-Cu-682 and ZnO/c-Cu-682 catalysts also remain the cubic morphology but some surface defects appear after CO reduction. Moreover, the ad-particles appearing on various ZnO/c-Cu-682 catalysts are verified to be ZnO in the HRTEM images, whose density and size grow up as the ZnO loading increase.

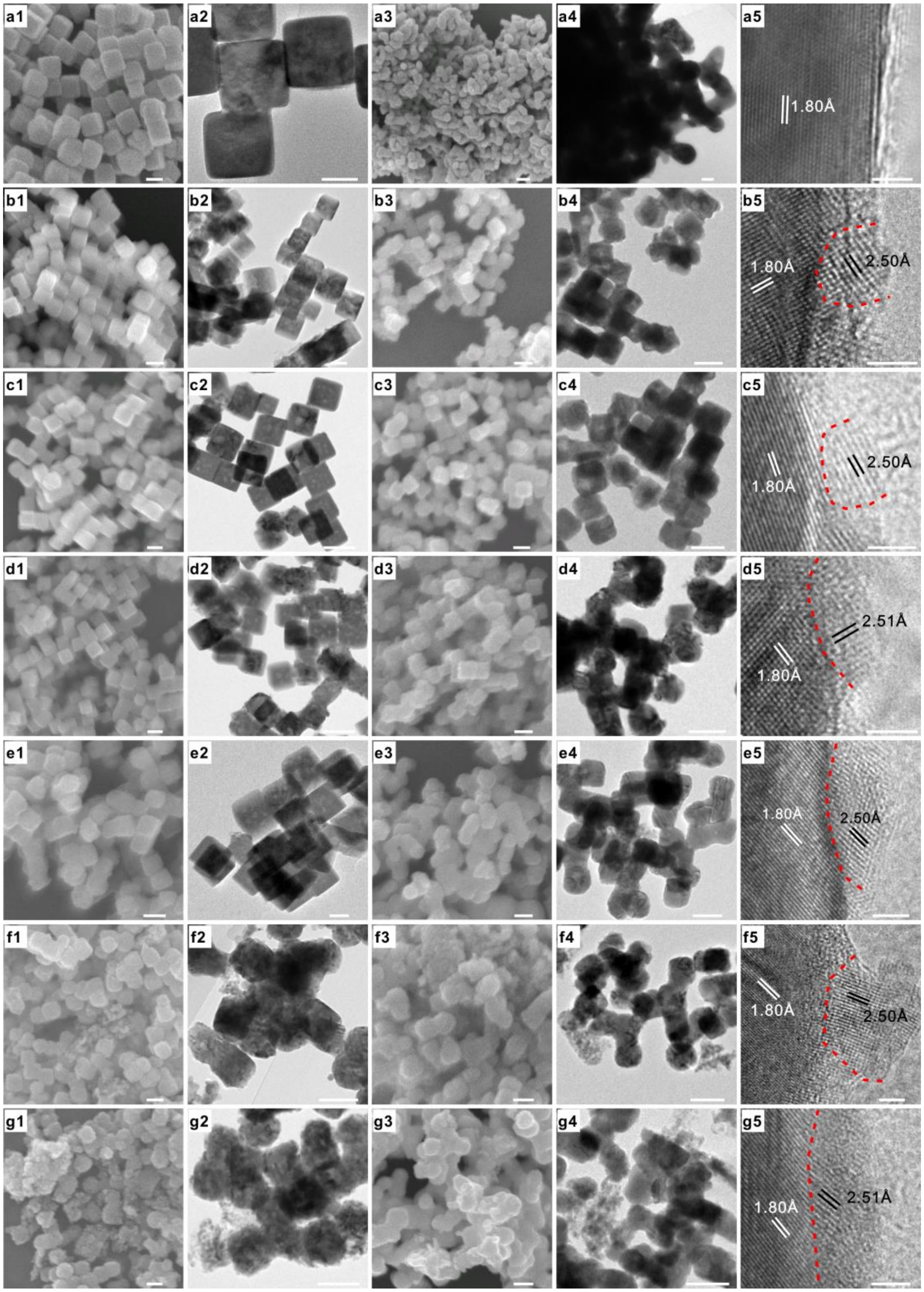

Supplementary Figure 5. The scale bars of (**a1-g1**), (**c2-g2**), (**b3-g3**), and (**b4-g4**) correspond to 100 nm, those of (**a2**), (**b2**), (**a3**), and (**a4**) correspond to 50 nm, and those of (**a5-g5**) correspond to 2 nm. SEM and TEM images of (**a1, a2**) c-Cu_2O-109, (**b1, b2**)

1%ZnO/c-Cu_2O-109, (**c1, c2**) 2%ZnO/c-Cu_2O-109, (**d1, d2**) 3%ZnO/c-Cu_2O-109, (**e1, e2**) 5%ZnO/c-Cu_2O-109, (**f1, f2**) 7%ZnO/c-Cu_2O-109, and (**g1, g2**) 9%ZnO/c-Cu_2O-109 catalysts; SEM, TEM, and HRTEM images (**a3-a5**) c-Cu-109, (**b3-b5**) 1%ZnO/c-Cu-109, (**c3-c5**) 2%ZnO/c-Cu-109, (**d3-d5**) 3%ZnO/c-Cu-109, (**e3-e5**) 5%ZnO/c-Cu-109, (**f3-f5**) 7%ZnO/c-Cu-109, and (**G3-G5**) 9%ZnO/c-Cu-109 catalysts. Lattice fringes of 1.80 and 2.50/2.51 Å respectively correspond to the spacing of Cu{200} (JCPDS card NO. 89-2838) and hexagonal ZnO{101} (JCPDS card NO 89-1397) crystal planes. The loading of ZnO on c-Cu_2O-109 NCs seldom affects the cubic morphology. After CO reduction, the acquired bare c-Cu-109 NCs are slight morphology change and serious agglomeration, which may arise from the finer size. However, such phenomenon can be alleviated on ZnO/c-Cu-109 NCs catalysts, indicating that the addition of ZnO can not only stabilize Cu NCs, but also resist Cu NCs to agglomerate. Moreover, the ad-particles appearing on various ZnO/c-Cu-109 catalysts are verified to be ZnO in the HRTEM images, whose density and size grow up as the ZnO loading increase.

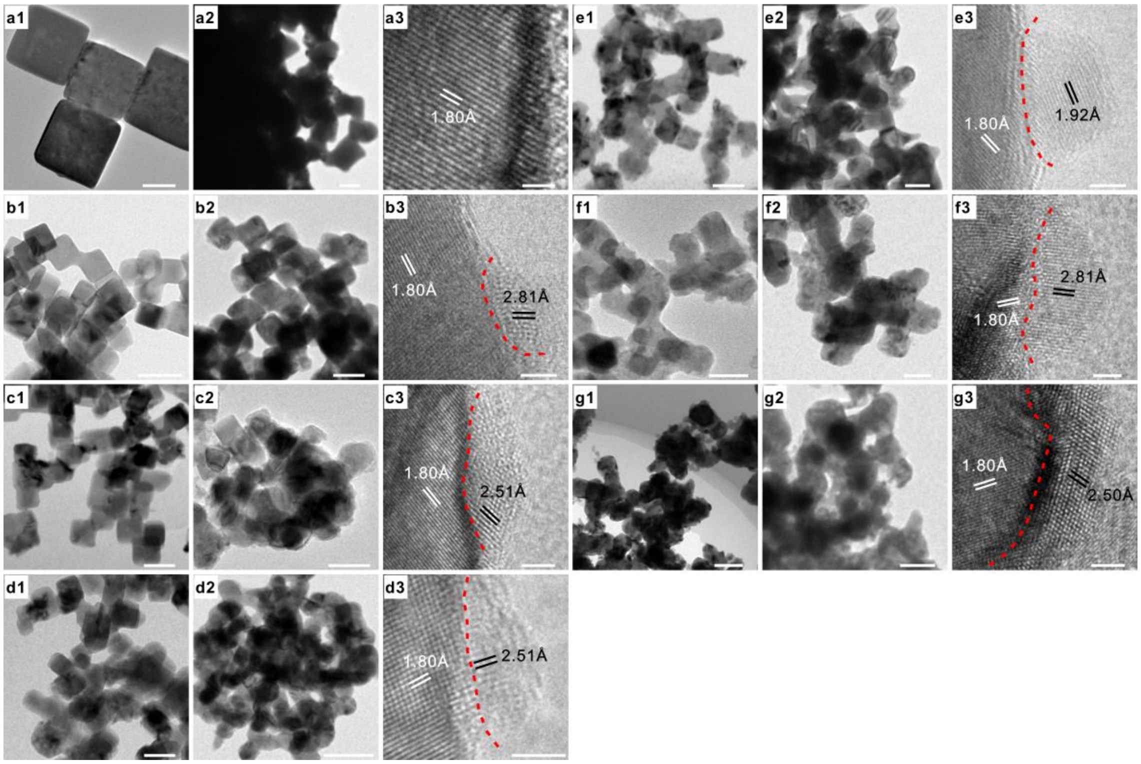

Supplementary Figure 6. The scale bars of (**b1-g1**) and (**a2-g2**) correspond to 50 nm, that of (**a1**) corresponds to 20 nm, those of (**b3-g3**) correspond to 2 nm, and that of (**a3**) corresponds to 1 nm. TEM images of (**a1**) c-Cu_2O-34, (**b1**) 1%ZnO/c-Cu_2O-34, (**c1**) 3%ZnO/c-Cu_2O-34, (**d1**) 5%ZnO/c-Cu_2O-34, (**e1**) 7%ZnO/c-Cu_2O-34, (**f1**) 9%ZnO/c-Cu_2O-34, and (**g1**) 11%ZnO/c-Cu_2O-34 catalysts; TEM and HRTEM images (**a2, a3**) c-Cu-34, (**b2, b3**) 1%ZnO/c-Cu-34, (**c2, c3**) 3%ZnO/c-Cu-34, (**d2, d3**) 5%ZnO/c-Cu-34, (**e2, e3**) 7%ZnO/c-Cu-34, (**f2, f3**) 9%ZnO/c-Cu-34, and (**g2, g3**) 11%ZnO/c-Cu-34 catalysts. Lattice fringes of 1.80, 1.91, 2.50/2.51, and 2.81 Å respectively correspond to the spacing of Cu{200} (JCPDS card NO. 89-2838), hexagonal ZnO{102}, ZnO{101}, and ZnO{100} (JCPDS card NO 89-1397) crystal planes. The loading of ZnO on c-Cu_2O-34 NCs seldom affects the cubic morphology. After CO reduction, the acquired bare c-Cu-34 NCs are slight morphology change and serious agglomeration, which may arise from the finer size. However, such phenomenon can be alleviated on ZnO/c-Cu-34 NCs catalysts, indicating that the addition of ZnO can

not only stabilize Cu NCs, but also resist Cu NCs to agglomerate. Moreover, the ad-particles appearing on various ZnO/c-Cu-34 catalysts are verified to be ZnO in the HRTEM images, whose density and size grow up as the ZnO loading increase.

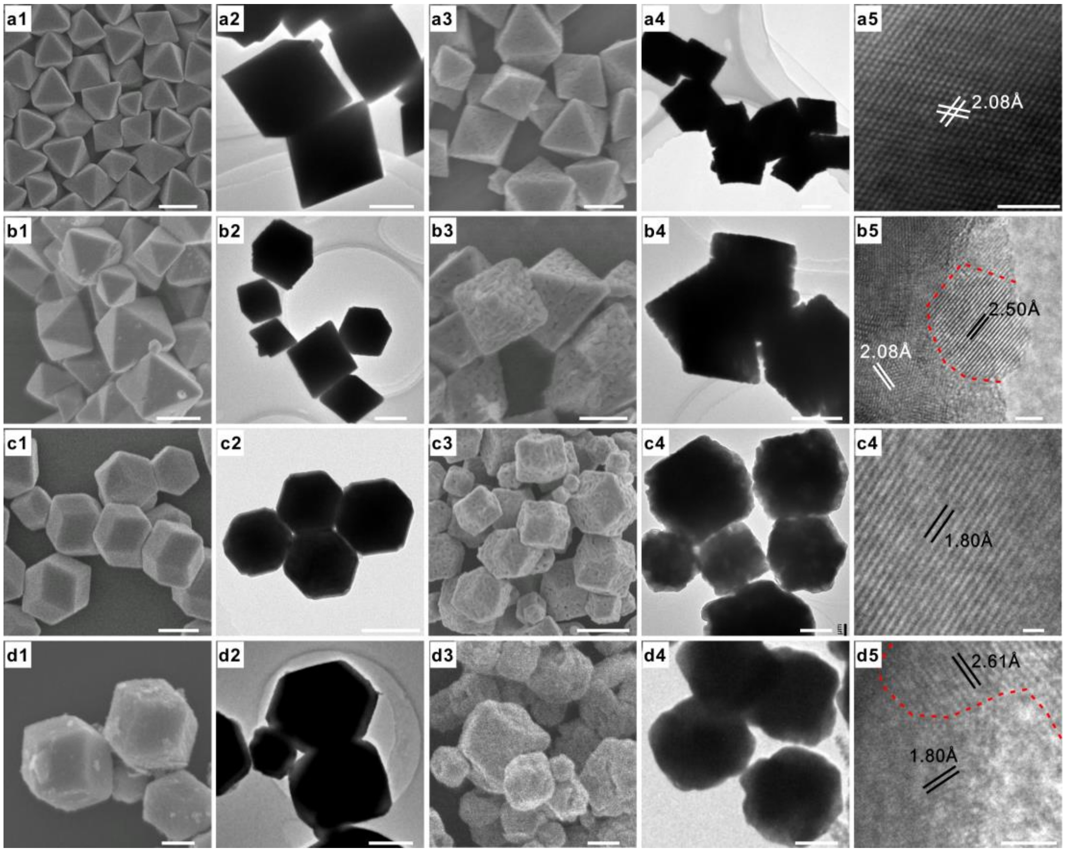

Supplementary Figure 7. The scale bar of (**c3**) corresponds to 1 μm, those of (**a1-d1**), (**a2-d2**), (**a3, b3, d3**), and (**a4-d4**) correspond to 500 nm, and those of (**a5-d5**) correspond to 2 nm. SEM and TEM images of (**a1, a2**) o-Cu_2O, (**b1, b2**) 1%ZnO/o-Cu_2O, (**c1, c2**) d-Cu_2O, and (**d1, d2**) 1%ZnO/d-Cu_2O catalysts; SEM, TEM, and HRTEM images (**a3-a5**) o-Cu, (**b3-b5**) 1%ZnO/o-Cu, (**c3-c5**) d-Cu, (**d3-d5**) 1%ZnO/d-Cu catalysts. Lattice fringes of 1.80, 2.50, and 2.61 Å respectively correspond to the spacing of Cu{200} (JCPDS card NO. 89-2838), hexagonal ZnO{101}, and ZnO{002} (JCPDS card NO 89-1397) crystal planes. The loading of ZnO on o-Cu_2O and d-Cu_2O NCs seldom affects their octahedral and rhombic dodecahedral morphologies and their surfaces attach with ad-particles. As well, the corresponding o&d-Cu and ZnO/o&d-Cu catalysts also remain the octahedral and rhombic dodecahedral morphologies but some surface defects appear after CO reduction. Moreover, the ad-particles appearing on 1%ZnO/o&d-Cu catalysts are verified to be ZnO in the HRTEM images.

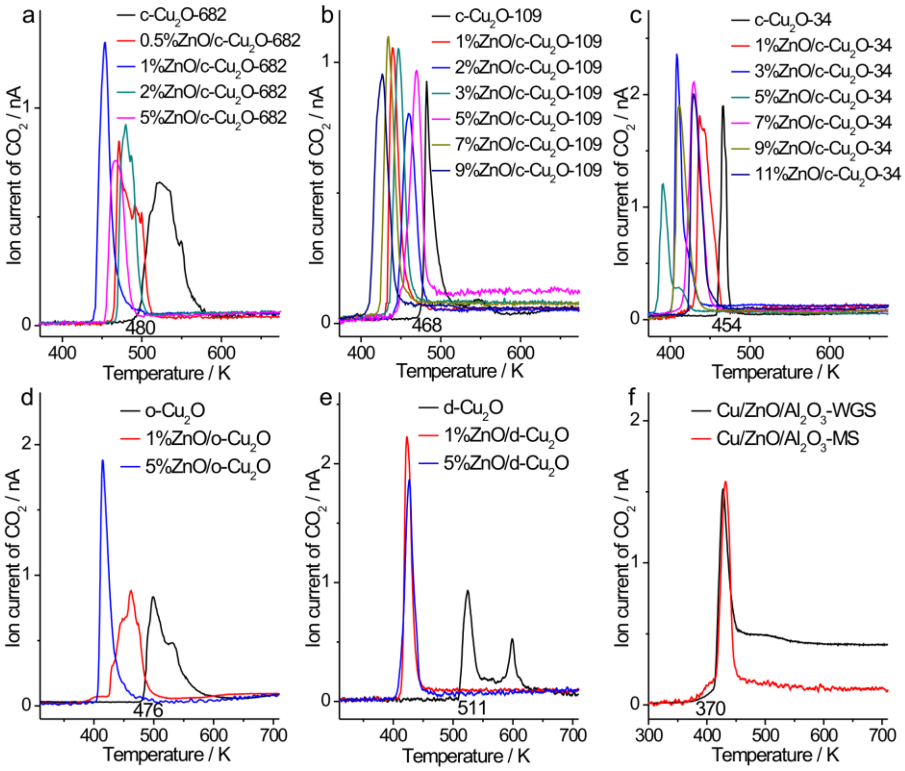

Supplementary Figure 8. CO-TPR profiles of (**a**) c-Cu_2O-682 NCs and various ZnO/c-Cu_2O-682 catalysts, (**b**) c-Cu_2O-109 NCs and various ZnO/c-Cu_2O-109 catalysts, (**c**) c-Cu_2O-34 NCs and various ZnO/c-Cu_2O-34 catalysts, (**d**) o-Cu_2O NCs and various ZnO/o-Cu_2O catalysts; (**e**) d-Cu_2O NCs and various ZnO/d-Cu_2O catalysts, and (**f**) commercial WGS and CO hydrogenation to methanol catalysts. According to the CO-TPR results, the initial reduction temperatures are 480, 468, 454, 476, 511, and 370 K for c-Cu_2O-682, c-Cu_2O-109, c-Cu_2O-34, o-Cu_2O, d-Cu_2O, and commercial catalysts, respectively. Therefore, a reduction process in 5% CO/Ar at 548 K for 2 h was chosen to fully reduce catalysts for c-Cu_2O-682, o-Cu_2O, and d-Cu_2O and their corresponding ZnO/Cu_2O catalysts, and in 5% CO/Ar at 473 K for 2 h was chosen to fully reduce catalysts for c-Cu_2O-109 and c-Cu_2O-34 and their corresponding ZnO/Cu_2O catalysts.

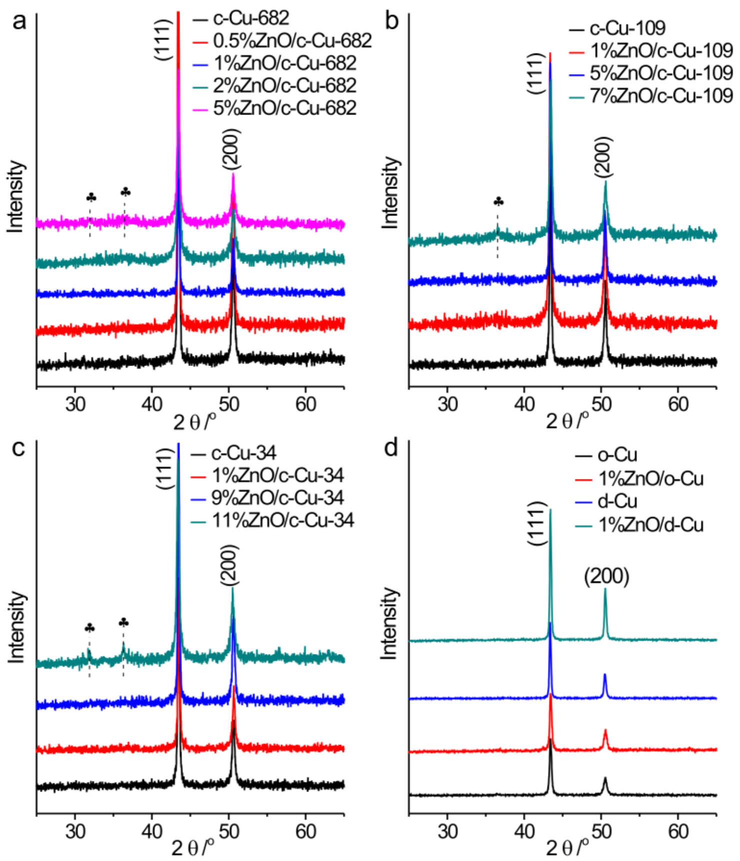

Supplementary Figure 9. XRD patterns of (**a**) c-Cu-682 NCs and various ZnO/c-Cu-682 catalysts, (**b**) c-Cu-109 NCs and various ZnO/c-Cu-109 catalysts, (**c**) c-Cu-34 NCs and various ZnO/c-Cu-34 catalysts, and (**d**) o&d-Cu NCs and 1%ZnO/o&d-Cu catalysts (♣: ZnO). Compared to the fresh catalysts (Supplementary Fig. 3), the crystal phases of Cu_2O completely transfer into those of metallic Cu after CO reduction, while the crystal phases of ZnO still exist in high ZnO loadings, indicating ZnO is not reduced by CO at chose conditions. In addition, the crystal phases of ZnO disappear on 9%ZnO/c-Cu-34 catalyst but not on 9%ZnO/c-Cu_2O-34 catalyst, suggesting that the reduction process may promote the redispersion of ZnO into smaller particles.

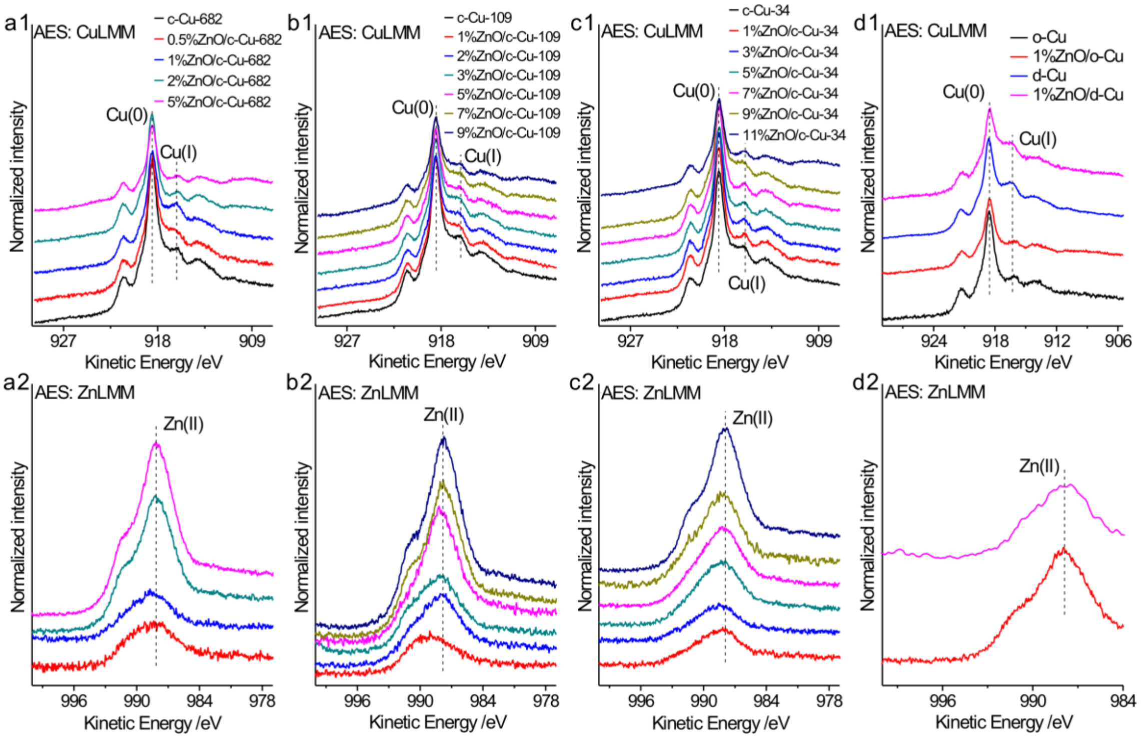

Supplementary Figure 10. CuLMM and ZnLMM AES spectra of (**a1, a2**) c-Cu-682 NCs and various ZnO/c-Cu-682 catalysts, (**b1, b2**) c-Cu-109 NCs and various ZnO/c-Cu-109 catalysts, (**c1, c2**) c-Cu-34 NCs and various ZnO/c-Cu-34 catalysts, and (**d1, d2**) o&d-Cu NCs and 1%ZnO/o&d-Cu catalysts. Cu LMM AES results confirm that the dominant Cu(0) accompanied by partial Cu(I) coexists in both Cu NCs and ZnO/Cu catalysts and Zn LMM AES spectra confirm Zn existence in the form of Zn(II).

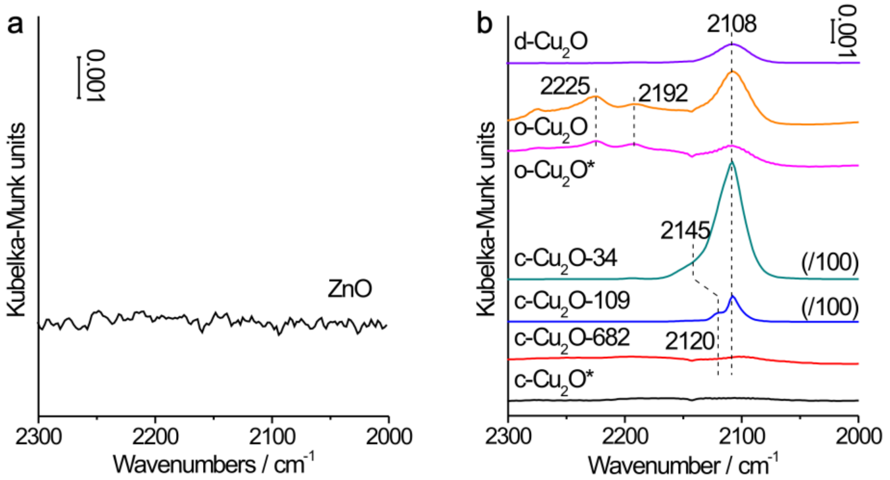

Supplementary Figure 11. In situ DRIFTS spectra of CO adsorption at 123 K of (**a**) ZnO nanoparticles and (**b**) various Cu_2O NCs. The particle sizes of c-Cu_2O^* and o-Cu_2O^* NCs are ca. 1000 nm. All DRIFTS spectra of Cu_2O NCs are same to our previous reports [17,18]. According to the previous assignments [17-19], the vibrational bands at 2108, 2120-2145, and 2192/2225 cm^{-1} can be ascribed to CO adsorbed on the terrace and defective Cu(I) sites, and CO_2 adsorbed on Cu(I) sites, respectively. In addition, pure ZnO nanoparticles were not observed to adsorb CO.

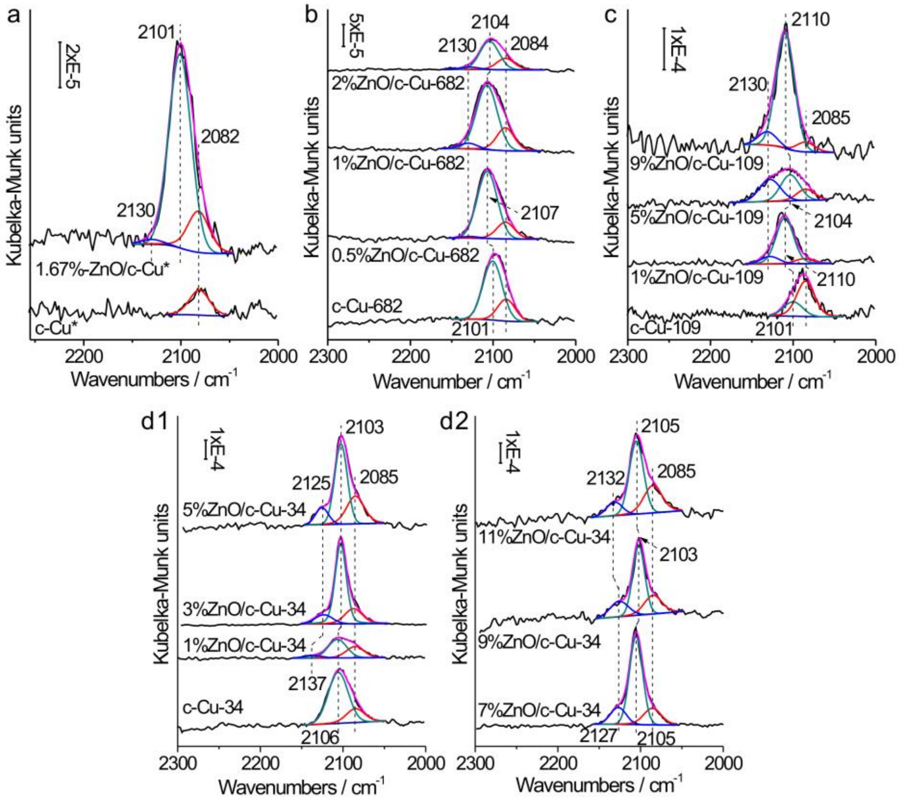

Supplementary Figure 12. In-situ DRIFTS spectra of CO adsorption at 123 K with peak-fitting results of (**a**) c-Cu* NCs and 1.67%-ZnO/c-Cu* catalyst, (**b**) c-Cu-682 NCs and various ZnO/c-Cu-682 catalysts, (**c**) c-Cu-109 NCs and various ZnO/c-Cu-109 catalysts, (**d1, d2**) c-Cu-34 NCs and various ZnO/c-Cu-34 catalysts. The red, light blue, and blue lines represent the fitted vibrational peaks of CO adsorbed on the Cu facets, defective sites of Cu, and Cu-ZnO interface, respectively. The particle sizes of c-Cu* and 1.67%-ZnO/c-Cu* NCs are ca. 1000 nm, same to our previous reports [17]. For bare c-Cu NCs, c-Cu* NCs only exhibit a vibrational band at 2082 cm^{-1}, ascribed to CO adsorbed at Cu(100) terrace sites [20,21]. As the decrease of c-Cu size, a new vibrational band at 2101~2106 cm^{-1} appears, which can be ascribed to CO adsorbed on the surface defective sites of c-Cu NCs [22]. The density of surface defective sites on various c-Cu NCs follows the order of c-Cu-34 > c-Cu-682 > c-Cu-109. c-Cu-109 NCs are finer than c-Cu-682 NCs but the density of surface defects is lower, which can be associated with different synthesis methods of various c-Cu_2O NCs. When ZnO nanoparticles were

supported on c-Cu NCs, in addition to the vibrational bands of CO adsorbed on Cu(100) terrace and surface defective sites at 2082-2085 and 2101-2110 cm^{-1}, an new vibrational band at 2125-2137 cm^{-1} was also observed, characteristic for CO adsorbed at the Cu(I) site [22]. Notably, the surface defective sites are absent for c-Cu^{*} but emerge for 1.67%-ZnO/c-Cu^{*}, indicating that ZnO supported on c-Cu^{*} NCs results in the formation of surface defects. Therefore, the surface defective sites on ZnO/c-Cu-682&109&34 catalysts not only inherit from the original Cu NCs, but also arise from the new generation during ZnO supported.

Supplementary Table 2. The fitting parameters of in situ DRIFTS of CO adsorption on various Cu and ZnO/Cu catalysts.

Catalysts	~2085 cm^{-1}			2101-2106 cm^{-1}			2130-2137 cm^{-1}		
	FWHM /cm^{-1}	L-G %	Areas	FWHM /cm^{-1}	L-G %	Areas	FWHM /cm^{-1}	L-G %	Areas
c-Cu-682	25	20	0.0026	27	0	0.0076	-	-	-
1%ZnO/ c-Cu-682	25	20	0.003	29	0	0.009	26	0	0.00035
2%ZnO/ c-Cu-682	25	20	0.0015	27	0	0.00365	26	0	0.000225
c-Cu-109	25	20	0.0025	27	0	0.001	-	-	-
1%ZnO/ c-Cu-109	25	20	0.00035	25	0	0.0031	26	0	0.0005
5%ZnO/ c-Cu-109	25	20	0.0008	27	0	0.0018	30	0	0.0017
9%ZnO/ c-Cu-109	25	20	0.0007	26	0	0.0077	26	0	0.0009
c-Cu-34	25	20	0.0023	28	0	0.0083	-	-	-
1%ZnO/ c-Cu-34	25	20	0.0018	26	0	0.0028	26	0	0.00037
3%ZnO/ c-Cu-34	25	20	0.0024	17	0	0.008	25	0	0.00133
5%ZnO/ c-Cu-34	25	20	0.0044	18	0	0.0083	19	0	0.0018
7%ZnO/ c-Cu-34	25	20	0.0025	19	0	0.009	22	0	0.00205
9%ZnO/ c-Cu-34	25	20	0.0028	17	0	0.0065	26	0	0.0022
11%ZnO/ c-Cu-34	25	20	0.0043	20	0	0.008	23	0	0.0017

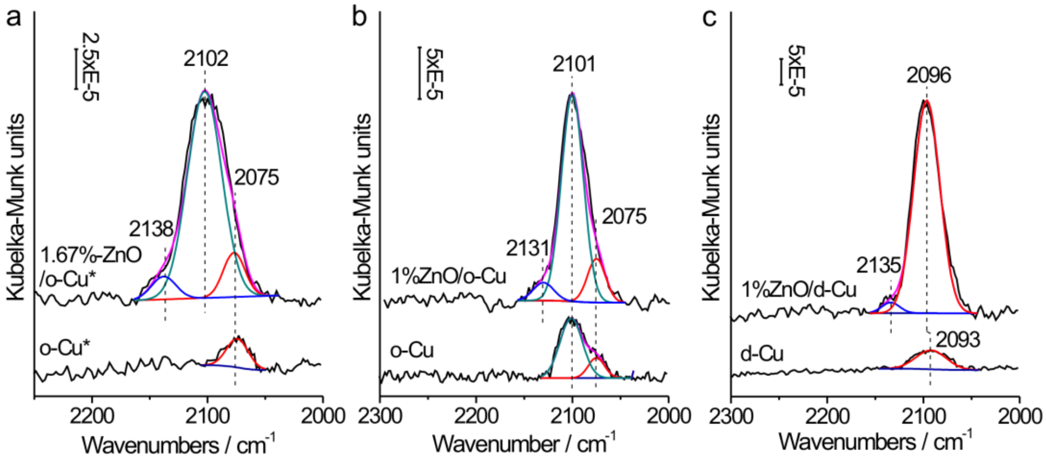

Supplementary Figure 13. In-situ DRIFTS spectra of CO adsorption at 123 K with peak-fitting results of (**a**) o-Cu* NCs and 1.67%-ZnO/o-Cu* catalyst, (**b**) o-Cu NCs and 1%ZnO/o-Cu catalyst, and (**c**) d-Cu NCs and 1%ZnO/d-Cu catalyst. The red, light blue, and blue lines represent the fitted vibrational peaks of CO adsorbed on the Cu facets, defective sites of Cu, and Cu-ZnO interface, respectively. The particle sizes of o-Cu* and 1.67%-ZnO/o-Cu* NCs are ca. 1000 nm, same to our previous reports [17]. For bare o-Cu NCs, o-Cu* NCs only exhibit a vibrational band at 2075 cm^{-1}, ascribed to CO adsorbed at Cu(111) terrace sites [20,23]. As the decrease of o-Cu size, a new vibrational band at 2101 cm^{-1} appears, which can be ascribed to CO adsorbed on the surface defective sites of c-Cu NCs [22]. When ZnO nanoparticles were supported on o-Cu NCs, in addition to the vibrational bands of CO adsorbed on Cu(111) terrace and surface defective sites at 2075 and 2101-2102 cm^{-1}, an new vibrational band at 2131-2138 cm^{-1} was also observed, characteristic for CO adsorbed at the Cu(I) site [22]. For d-Cu NCs, bare d-Cu NCs exhibit only a vibrational band at 2093 cm^{-1}, ascribed to CO adsorbed on Cu(110) terrace sites [20,24], while an additional vibrational band at 2135 cm^{-1} was observed on 1%ZnO/d-Cu catalyst, also characteristic for CO adsorbed at the Cu(I) site [22]. These suggest that the surface of d-Cu NCs is relatively more stable during CO reduction process.

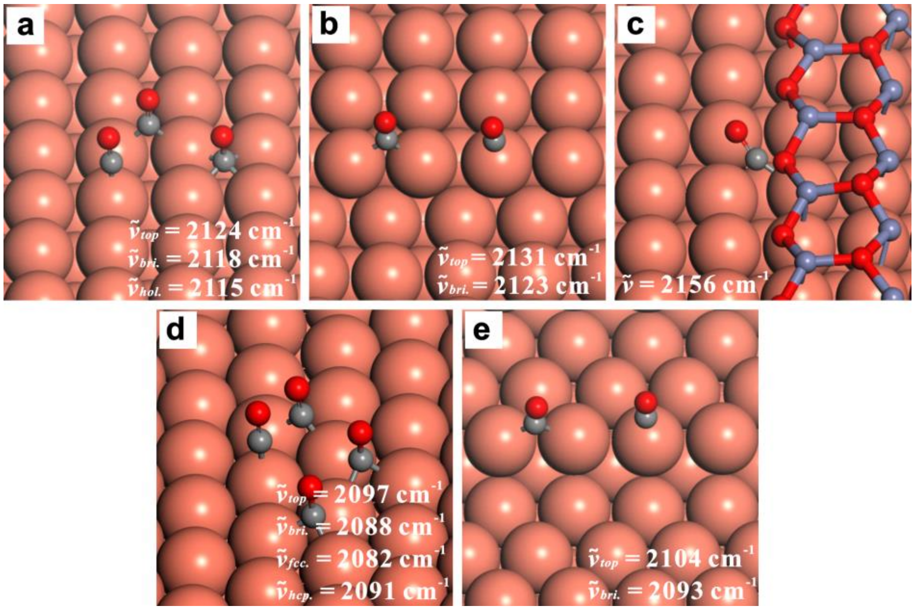

Supplementary Figure 14. Optimized structures of stable CO species adsorbed on (**a**) Cu(100), (**b**) Cu(611), (**c**) ZnO/Cu(100), (**d**) Cu(111), and (**e**) Cu (211) surfaces in different CO adsorption forms with the C=O stretching frequencies. Red, gray, and pink balls represent O, C, and Cu atoms, respectively. According to the reported literatures [6,25], Cu(611) and Cu(211) surfaces can be employed to replace the defective Cu(100) and Cu(111) surfaces, respectively.

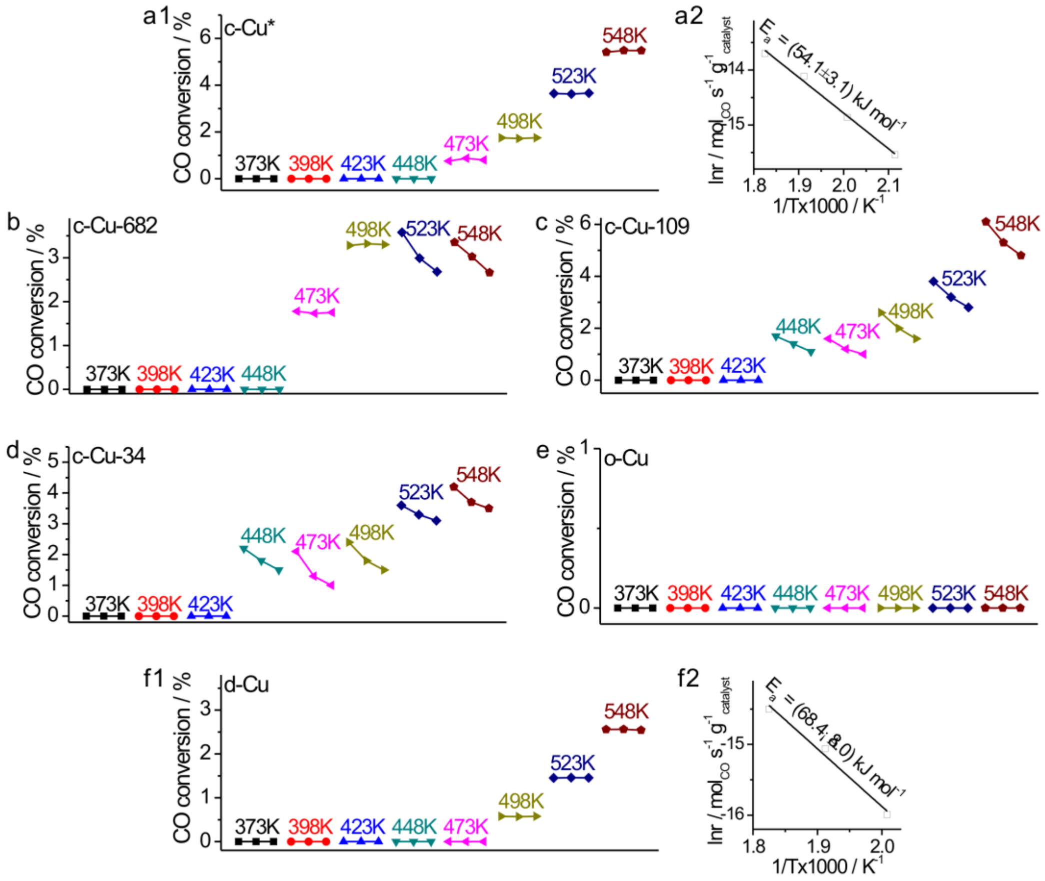

Supplementary Figure 15. Catalytic performance with three measurement points at each temperature and corresponding Arrhenius plots of **(a1, a2)** c-Cu*, **(b)** c-Cu-682, **(c)** c-Cu-109, **(d)** c-Cu-34, **(e)** o-Cu, and (**f1, f2**) d-Cu NCs in the WGS reaction. The particle size of c-Cu* is ca. 1000 nm. The catalytic performance of various Cu morphologies in catalyzing the WGS reaction follows the order of c-Cu > d-Cu > o-Cu, in consistent with our previous result [17]. Moreover, as the size shrinkage of c-Cu NCs, the catalytic performance is slightly increasing while their stabilities are gradually poor.

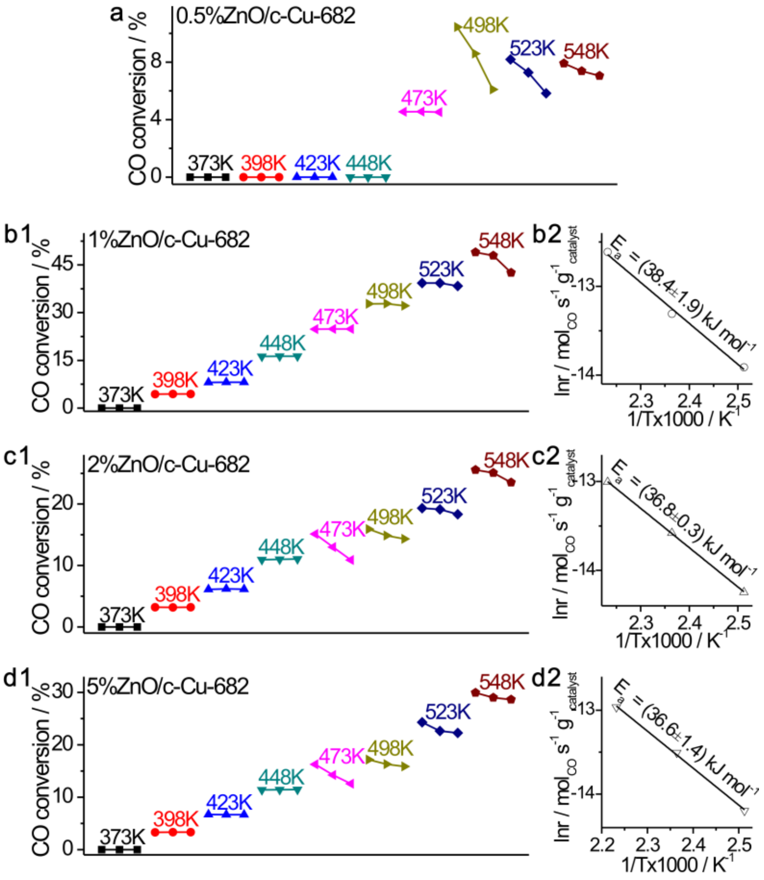

Supplementary Figure 16. (**a**) Catalytic performance with three measurement points at each temperature of 0.5%ZnO/c-Cu-682 catalyst in the WGS reaction; Catalytic performance with three measurement points at each temperature and the corresponding Arrhenius plots of (**b1, b2**) 1%ZnO/c-Cu-682, (**c1, c2**) 2%ZnO/c-Cu-682, and (**d1, d2**) 5%ZnO/c-Cu-682 catalysts in the WGS reaction. Compared to c-Cu-682 NCs, ZnO supported on c-Cu-682 NCs can significantly promote both the activity and stability. Among them, 1%ZnO/c-Cu-682 catalyst is not only the relatively most active, but also the relatively most stable catalyst. The Arrhenius plots with stable CO conversions that are below 20% are used to calculate E_a, whose values are all similar for ZnO/c-Cu-682 catalysts, suggesting that they involve with the same catalytic active sites in WGS reaction.

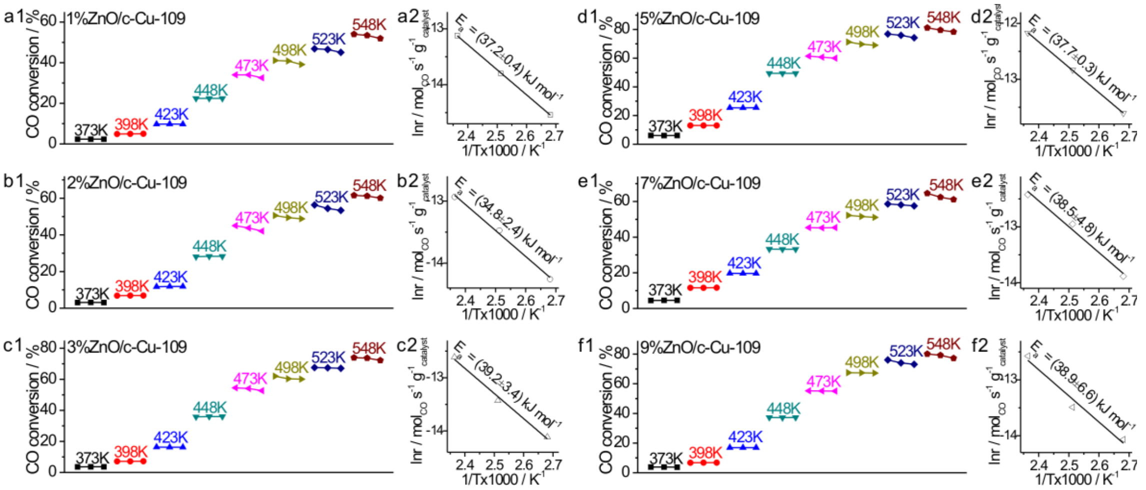

Supplementary Figure 17. Catalytic performance with three measurement points at each temperature and the corresponding Arrhenius plots of (**a1, a2**) 1%ZnO/c-Cu-109, (**b1, b2**) 2%ZnO/c-Cu-109, (**c1, c2**) 3%ZnO/c-Cu-109, (**d1, d2**) 5%ZnO/c-Cu-109, (**e1, e2**) 7%ZnO/c-Cu-109, and (**f1, f2**) 9%ZnO/c-Cu-109 catalysts in the WGS reaction. Compared to c-Cu-109 NCs, ZnO supported on c-Cu-109 NCs can significantly promote both the activity and stability. Among them, 5%ZnO/c-Cu-109 catalyst is not only the relatively most active, but also the relatively most stable catalyst. The Arrhenius plots with stable CO conversions that are below 20% are used to calculate E_a, whose values are all similar for ZnO/c-Cu-109 catalysts, suggesting that they involve with the same catalytic active sites in WGS reaction.

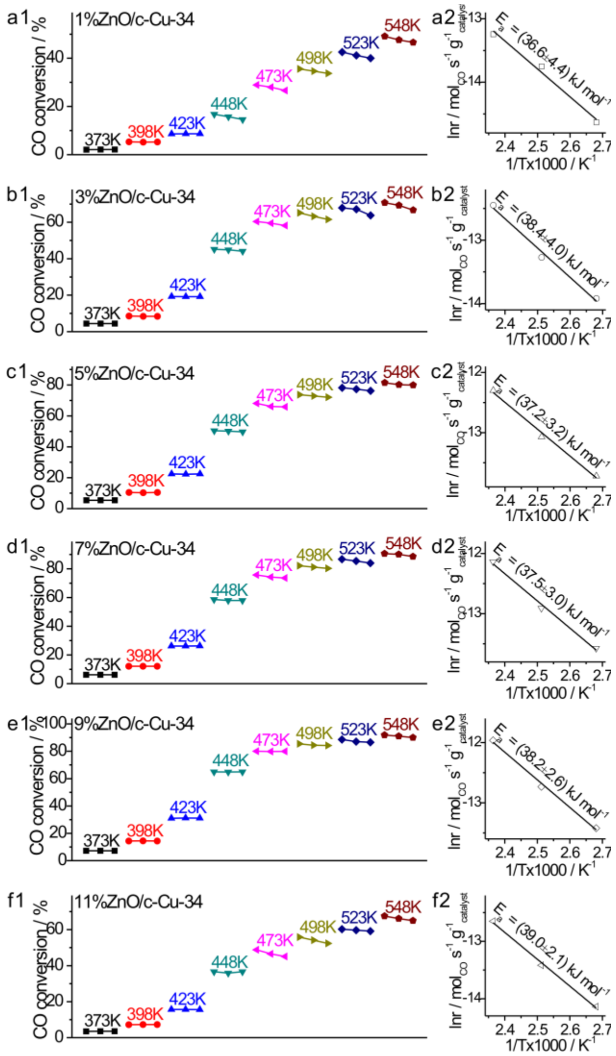

Supplementary Figure 18. Catalytic performance with three measurement points at each temperature and the corresponding Arrhenius plots of (**a1, a2**) 1%ZnO/c-Cu-34, (**b1, b2**) 3%ZnO/c-Cu-34, (**c1, c2**) 5%ZnO/c-Cu-34, (**d1, d2**) 7%ZnO/c-Cu-34, (**e1, e2**) 9%ZnO/c-Cu-34, and (**f1, f2**) 11%ZnO/c-Cu-34 catalysts in the WGS reaction. Compared to c-Cu-34 NCs, ZnO supported on c-Cu-34 NCs can significantly promote both the activity and stability. Among them, 9%ZnO/c-Cu-34 catalyst is not only the relatively most active, but also the relatively most stable catalyst. The Arrhenius plots with stable CO conversions that are below 20% are used to calculate E_a, whose values are all similar for ZnO/c-Cu-34 catalysts, suggesting that they involve with the same catalytic active sites in WGS reaction.

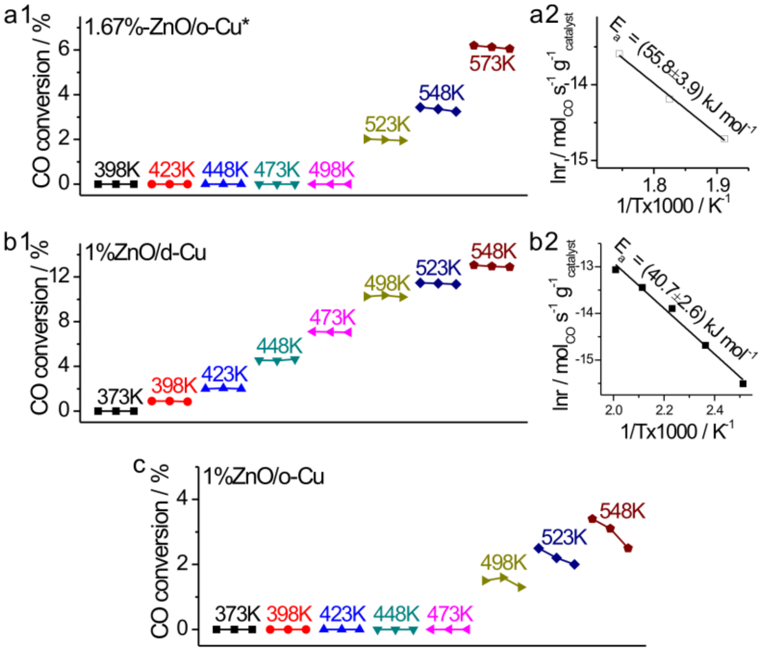

Supplementary Figure 19. Catalytic performance with three measurement points at each temperature and the corresponding Arrhenius plots of (**a1, a2**) 1.67%-ZnO/o-Cu* and (**b1, b2**) 1%ZnO/d-Cu catalysts in the WGS reaction; (**c**) Catalytic performance with three measurement points at each temperature of 1%ZnO/o-Cu catalyst in the WGS reaction. The particle size of 1.67%-ZnO/o-Cu* is ca. 1000 nm. Compared to bare o-Cu NCs, ZnO supported on o-Cu NCs can slightly promote the activity. As the size decrease of o-Cu in ZnO/o-Cu catalyst, the catalytic activity increases but the catalytic stability is poor. Compared to d-Cu NCs, ZnO supported on d-Cu NCs can promote both the activity. The Arrhenius plots with stable CO conversions that are below 20% are used to calculate E_a, whose value is lower than corresponding d-Cu NCs (Supplementary Fig. 15), indicating the intrinsic activity of the WGS reaction catalyzed by ZnO/d-Cu is more active than d-Cu NCs.

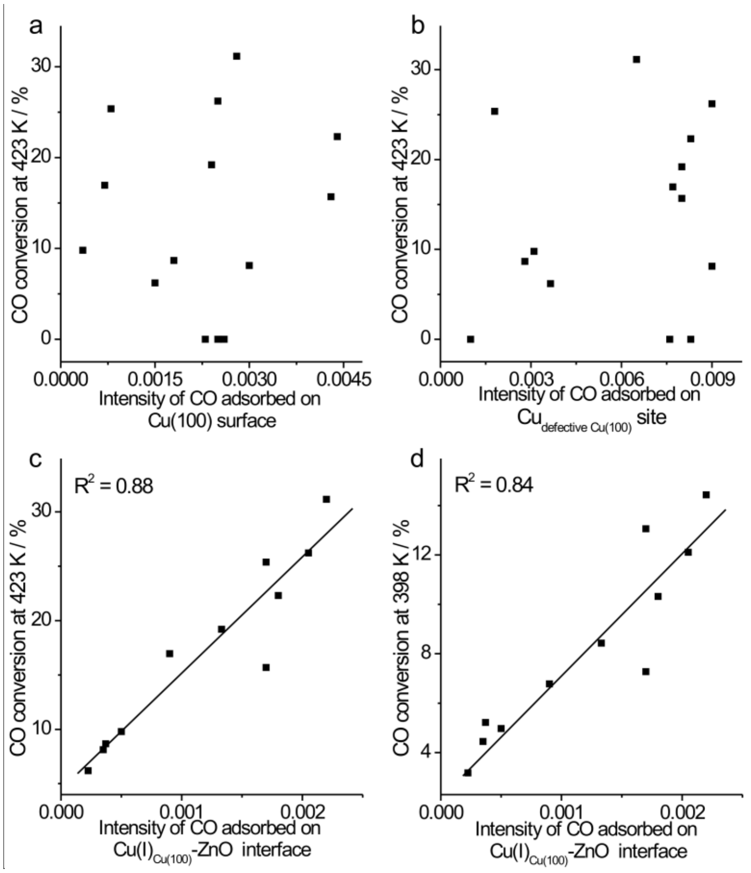

Supplementary Figure 20. The linear relation of CO conversion at 423 K in the WGS reaction with (**a**) the fitting area of CO adsorbed on Cu(100) facet, (**b**) the fitting area of CO adsorbed on $Cu_{defective\ Cu(100)}$ site, and (**c**) the fitting area of CO adsorbed on $Cu(I)_{Cu(100)}$-ZnO interface site; (**d**) The linear relation of CO conversion at 398 K in the WGS reaction with the fitting area of CO adsorbed on $Cu(I)_{Cu(100)}$-ZnO interface. The results clearly reveal that the catalytic performance of various ZnO/c-Cu catalysts in catalyzing WGS reaction at and below 423 K is proportional to the amount of CO adsorbed at the Cu(I) site of Cu-O-Zn interface, suggesting the low-temperature WGS reaction proceeds at the Cu-ZnO interface of ZnO/c-Cu catalysts. Furthermore, the reaction temperatures above 423 K are not considered due to the emergence of the catalytic activity of c-Cu NCs (Supplementary Fig. 15).

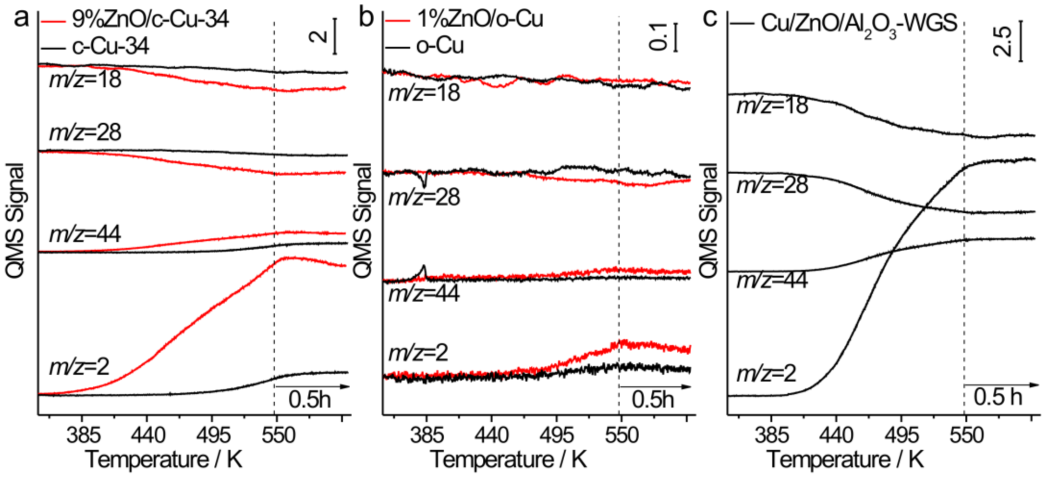

Supplementary Figure 21. CO+H_2O-TPRS spectra of WGS reaction over (**a**) c-Cu-34 and 9%ZnO/c-Cu-34 catalyst, (**b**) o-Cu and 1%ZnO/o-Cu catalyst, and (**c**) commercial Cu/ZnO/Al_2O_3 WGS catalyst in 5% CO and 319 K water vapor (water vapor pressure: 10.094 kPa) balanced with Ar. Different from bare Cu NCs, the H_2 and CO_2 productions are simultaneous for all ZnO/Cu catalysts, indicating that WGS reaction catalyzed by ZnO/Cu catalysts and WGS reaction catalyzed by Cu NCs involves different catalytically active sites. 9%ZnO/c-Cu-34 catalyst exhibits more efficient property than 1%ZnO/o-Cu catalyst, the initial CO_2 and H_2 productions for 9%ZnO/c-Cu-34 catalyst occur at ~358 K that is even more facilely than commercial Cu/ZnO/Al_2O_3 WGS catalyst with the initial CO_2 and H_2 productions at ~398 K. These results further confirm that acquired 9%ZnO/c-Cu-34 catalyst is more active than commercial Cu/ZnO/Al_2O_3 WGS catalyst.

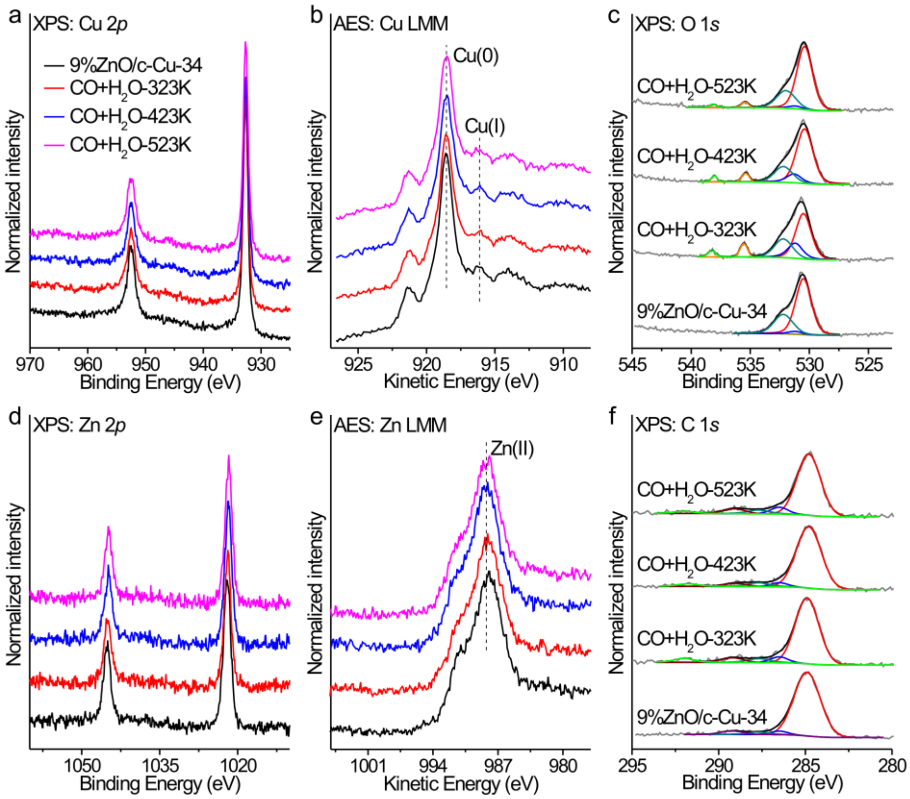

Supplementary Figure 22. (**a**) Cu 2*p* XPS spectra, (**b**) Cu LMM AES spectra, (**c**) O 1*s* XPS spectra with peak-fitting results, (**d**) Zn 2*p* XPS spectra, (**e**) Zn LMM AES spectra, and (**f**) C 1*s* XPS with peak-fitting results of fresh 9%ZnO/c-Cu-34 and corresponding 9%ZnO/c-Cu-34 subjected to 0.33 mbar CO + 0.67 mbar H_2O at different temperatures for 1 h. Cu 2*p* XPS and Cu LMM AES spectra demonstrate that Cu(0) is the dominant component in 9%ZnO/c-Cu-34 catalyst, accompanied by weak Cu(I) signal. Corresponding Zn 2*p* XPS and Zn LMM AES spectra demonstrate that zinc is exclusively composed of Zn(II). As the variations of pretreatment conditions, both Cu and Zn speciation of 9%ZnO/c-Cu-34 catalyst do not vary. O 1*s* XPS spectra show five types of oxygenous components at 530.5, 521.2, 532.3, 535.6, and 538.4 eV, which are ascribed to ZnO+Cu_xO, hydroxyl groups, oxygenates, gaseous H_2O, and gaseous CO, respectively. C 1*s* XPS spectra show five types of carbonaceous components at 284.8, 286.5, 287.7, 289.1, and 291.8 eV, which are ascribed to adventitious carbon, formate, carboxylate, carbonate, and gaseous CO, respectively.

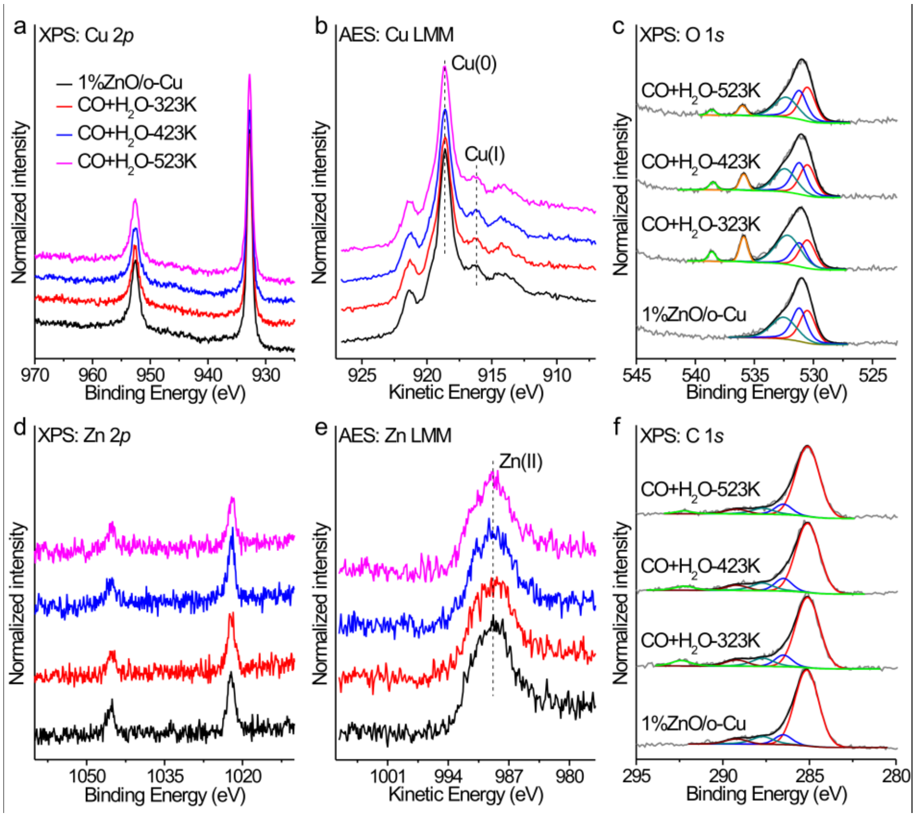

Supplementary Figure 23. (**a**) Cu 2*p* XPS spectra, (**b**) Cu LMM AES spectra, (**c**) O 1*s* XPS spectra with peak-fitting results, (**d**) Zn 2*p* XPS spectra, (**e**) Zn LMM AES spectra, and (**F**) C 1*s* XPS with peak-fitting results of fresh 1%ZnO/o-Cu and corresponding 1%ZnO/o-Cu subjected to 0.33 mbar CO + 0.67 mbar H_2O at different temperatures for 1 h. Cu 2*p* XPS and Cu LMM AES spectra demonstrate that Cu(0) is the dominant component in 1%ZnO/o-Cu catalyst, accompanied by weak Cu(I) signal. Corresponding Zn 2*p* XPS and Zn LMM AES spectra demonstrate that zinc is exclusively composed of Zn(II). As the variations of pretreatment conditions, both Cu and Zn speciation of 1%ZnO/o-Cu catalyst do not vary. O 1*s* XPS spectra show five types of oxygenous components at 530.5, 521.2, 532.3, 535.9, and 538.5 eV, which are ascribed to ZnO+Cu_xO, hydroxyl groups, oxygenates, gaseous H_2O, and gaseous CO, respectively. C 1*s* XPS spectra show five types of carbonaceous components at 284.8, 286.5, 287.7, 289.1, and 292.1 eV, which are ascribed to adventitious carbon, formate, carboxylate, carbonate, and gaseous CO, respectively.

Supplementary Table 3. The integrated peak areas of different O 1*s* and C 1*s* components on fresh ZnO/Cu and corresponding ZnO/Cu subjected to 0.33 mbar CO+0.67 mbar H_2O at different temperatures for 1 h acquired from the peak-fitting spectra of O 1*s* and C 1*s* XPS spectra in Supplementary Figs. 22 and 23.

	O 1*s* Binding Energy (eV)				
	530.5	531.2	532.3	535.7	538.5
9%ZnO/c-Cu-34	1.90	0.10	0.79	/	/
CO+H_2O-323K	1.54	0.55	0.73	0.19	0.10
CO+H_2O-423K	1.96	0.32	0.62	0.12	0.07
CO+H_2O-523K	2.14	0.10	0.72	0.08	0.05
1%ZnO/o-Cu	0.89	0.99	0.95	/	/
CO+H_2O-323K	0.77	0.70	1.27	0.32	0.12
CO+H_2O-423K	0.85	0.93	0.89	0.21	0.10
CO+H_2O-523K	0.93	0.85	0.80	0.13	0.07

	C 1*s* Binding Energy (eV)			
	286.5	287.7	289.1	292.1
9%ZnO/c-Cu-34	0.10	0.10	0.11	/
CO+H_2O-323K	0.16	0.16	0.15	0.14
CO+H_2O-423K	0.11	0.16	0.10	0.09
CO+H_2O-523K	0.19	0.16	0.16	0.08
1%ZnO/o-Cu	0.26	0.30	0.15	/
CO+H_2O-323K	0.31	0.32	0.19	0.18
CO+H_2O-423K	0.34	0.30	0.16	0.13
CO+H_2O-523K	0.28	0.24	0.14	0.08

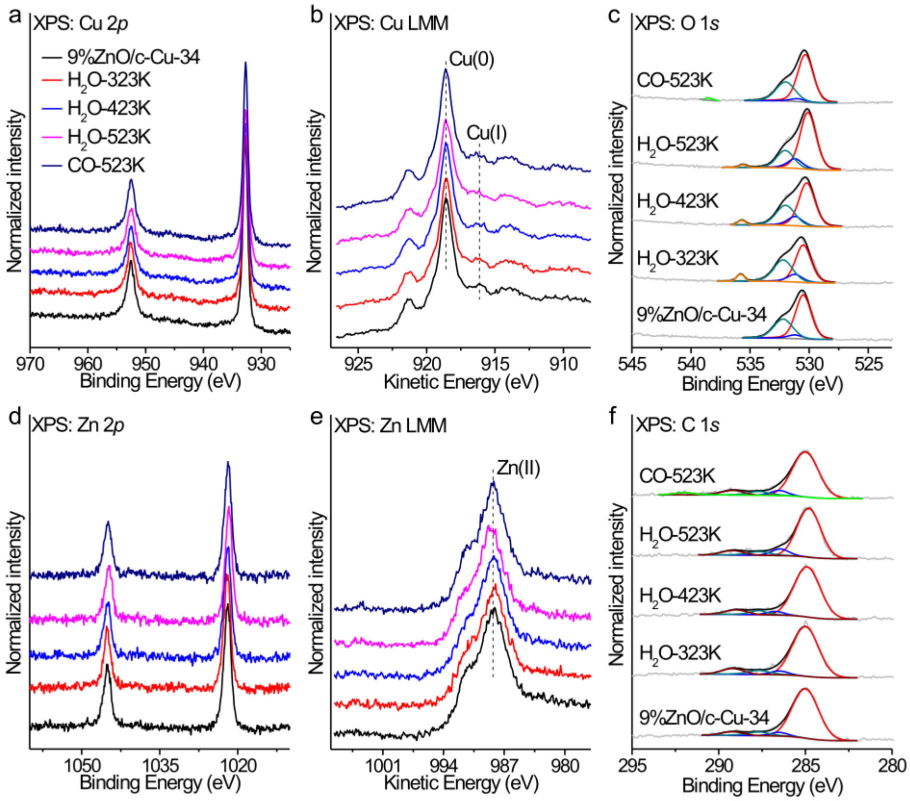

Supplementary Figure 24. (**a**) Cu 2*p* XPS spectra, (**b**) Cu LMM AES spectra, (**c**) O 1*s* XPS spectra with peak-fitting results, (**d**) Zn 2*p* XPS spectra, (**e**) Zn LMM AES spectra, and (**f**) C 1*s* XPS with peak-fitting results of fresh 9%ZnO/c-Cu-34 and corresponding 9%ZnO/c-Cu-34 subjected to 1 mbar H_2O at different temperatures and subsequently 1 mbar CO at 523 K for 1 h. As the variations of 9%ZnO/c-Cu-34 catalyst suffering from firstly water and then to CO, the surface structures of both Cu and Zn speciation do not vary, in consistent with the NAP-XPS results of CO+H_2O experiments, further confirming the WGS reaction catalyzed by the c-Cu-ZnO interface should not follow the redox mechanism.

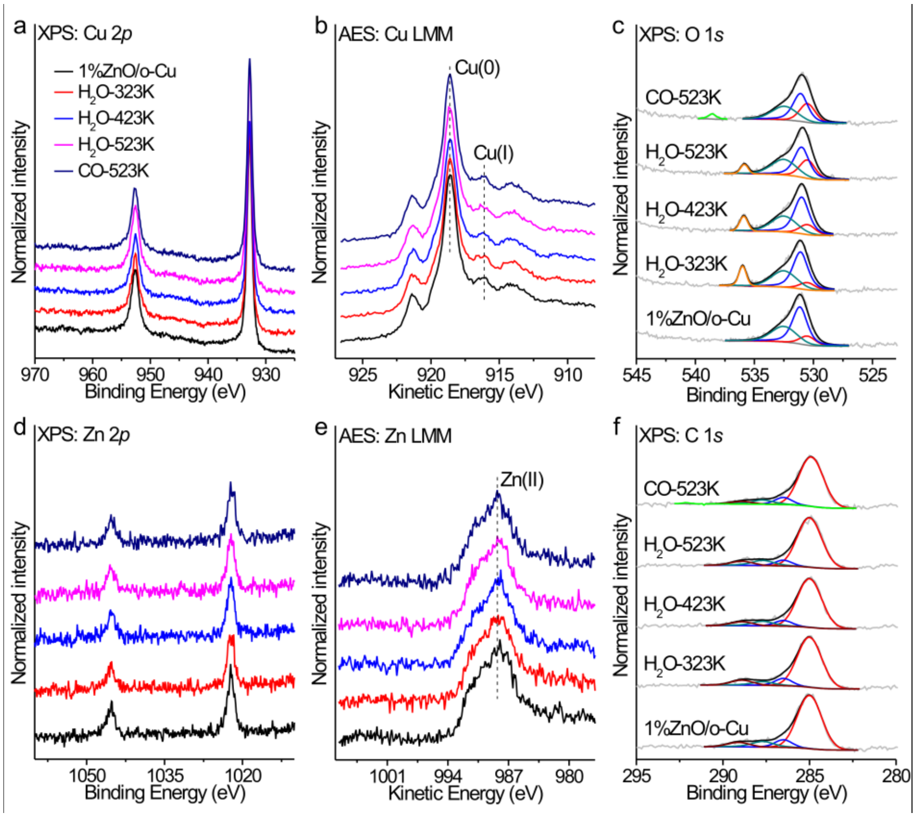

Supplementary Figure 25. (**a**) Cu 2*p* XPS spectra, (**b**) Cu LMM AES spectra, (**c**) O 1*s* XPS spectra with peak-fitting results, (**d**) Zn 2*p* XPS spectra, (**e**) Zn LMM AES spectra, and (**f**) C 1*s* XPS with peak-fitting results of fresh 1%ZnO/o-Cu and corresponding 1%ZnO/o-Cu subjected to 1 mbar H_2O at different temperatures and subsequently 1 mbar CO at 523 K for 1 h. As the variations of 1%ZnO/o-Cu catalyst suffering from firstly water and then to CO, the surface structures of both Cu and Zn speciation do not vary, in consistent with the NAP-XPS results of CO+H_2O experiments, further confirming the WGS reaction catalyzed by the o-Cu-ZnO interface should not follow the redox mechanism.

Supplementary Table 4. The integrated peak areas of different O 1*s* and C 1*s* components on fresh ZnO/Cu and corresponding ZnO/Cu subjected to 1 mbar H_2O at different temperatures and subsequently 1 mbar CO at 523 K for 1 h acquired from the peak-fitting spectra of O 1*s* and C 1*s* XPS spectra in Supplementary Figs. 24 and 25.

	O 1*s* Binding Energy (eV)				
	530.5	531.2	532.3	535.7	538.5
9%ZnO/c-Cu-34	2.26	0.21	1.18	/	/
H_2O-323K	1.92	0.43	1.33	0.15	/
H_2O-423K	2.28	0.51	1.24	0.10	/
H_2O-523K	2.99	0.53	0.97	0.06	/
CO-523K	2.44	0.16	1.18	/	0.07
1%ZnO/o-Cu	0.30	1.32	0.82	/	/
H_2O-323K	0.24	1.31	0.85	0.30	/
H_2O-423K	0.33	1.30	0.83	0.22	/
H_2O-523K	0.60	1.07	0.81	0.13	/
CO-523K	0.62	0.99	0.71	/	0.11

	C 1*s* Binding Energy (eV)			
	286.5	287.7	289.1	292.1
9%ZnO/c-Cu-34	0.11	0.16	0.12	/
H_2O-323K	0.12	0.18	0.20	/
H_2O-423K	0.11	0.14	0.16	/
H_2O-523K	0.20	0.16	0.16	/
CO-523K	0.15	0.17	0.16	0.08
1%ZnO/o-Cu	0.26	0.28	0.18	/
H_2O-323K	0.25	0.19	0.20	/
H_2O-423K	0.19	0.23	0.15	/
H_2O-523K	0.19	0.26	0.17	/
CO-523K	0.24	0.21	0.10	0.02

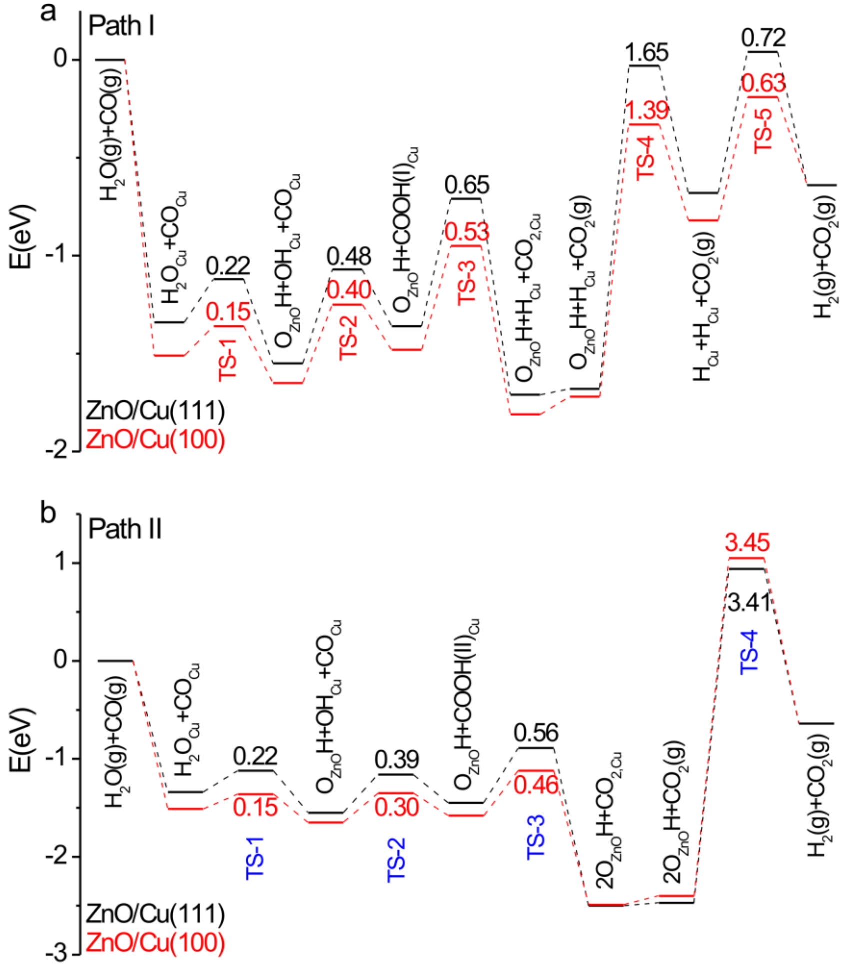

Supplementary Figure 26. The energy profiles of each elementary step in WGS reaction involved with (**a**) Path I and (**b**) Path II catalyzed by fresh ZnO/Cu(111) and ZnO/Cu(100).

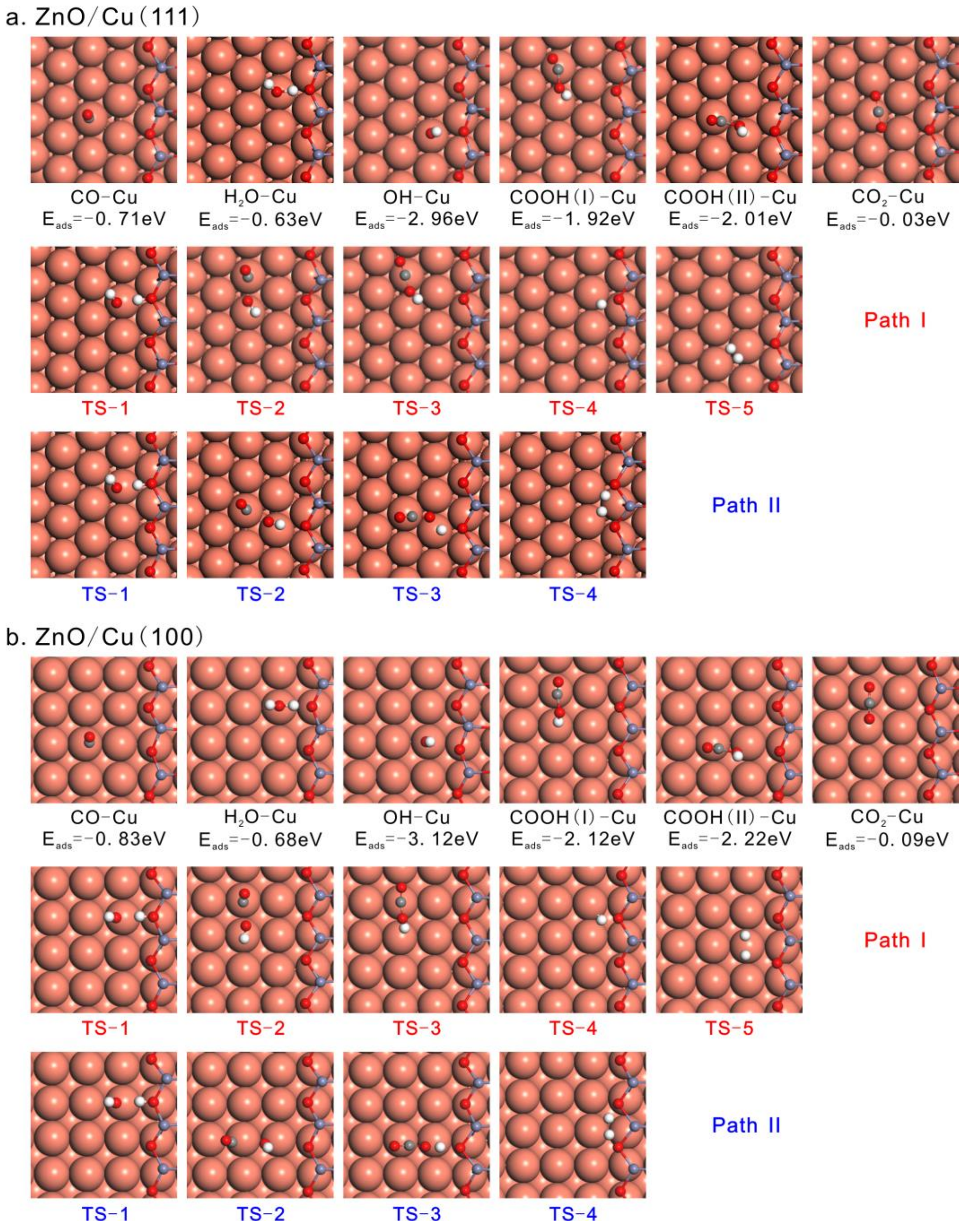

Supplementary Figure 27. The structures with the adsorption energies (E_{ads}) of various adsorption species and the structures at the different transition states in Supplementary Figure 26 of fresh (**a**) ZnO/Cu(111) and (**b**) ZnO/Cu(100) surfaces in catalyzing WGS reaction. The reddish-orange, purple, red, grey, and white spheres represent Cu, Zn, O, C, and H atoms, respectively.

Supplementary Table 5. Reaction energy (E_r) and activation energy (E_a) of each elementary step of WGS reaction involved with two reaction paths on fresh ZnO/Cu(111) and ZnO/Cu(100) surfaces.

Elementary reactions		ZnO/Cu(111)		ZnO/Cu(100)	
		E_r (eV)	E_a (eV)	E_r (eV)	E_a (eV)
$H_2O(g) + Cu_{ZnO\text{-}Cu} \rightarrow H_2O_{Cu}$		-0.63		-0.68	
$CO(g) + Cu_{ZnO\text{-}Cu} \rightarrow CO_{Cu}$		-0.71		-0.83	
$H_2O_{Cu} \rightarrow OH_{Cu} + O_{ZnO}H$		-0.21	0.22	-0.14	0.15
Path I	$CO_{Cu} + OH_{Cu} \rightarrow COOH(I)_{Cu}$	0.19	0.48	0.17	0.40
	$COOH(I)_{Cu} \rightarrow CO_{2,Cu} + H_{Cu}$	-0.35	0.65	-0.33	0.53
	$CO_{2,Cu} \rightarrow CO_2(g)$	0.03		0.09	
	$O_{ZnO}H \rightarrow O_{ZnO} + H_{Cu}$	1.00	1.65	0.90	1.39
	$H_{Cu} + H_{Cu} \rightarrow H_2(g)$	0.04	0.72	0.18	0.63
Path II	$CO_{Cu} + OH_{Cu} \rightarrow COOH(II)_{Cu}$	0.10	0.39	0.07	0.30
	$COOH(II)_{Cu} \rightarrow CO_{2,Cu} + O_{ZnO}H$	-1.05	0.56	-0.91	0.46
	$CO_{2,Cu} \rightarrow CO_2(g)$	0.03		0.09	
	$O_{ZnO}H + O_{ZnO}H \rightarrow H_2(g) + 2 O_{ZnO}$	1.83	3.41	1.76	3.45

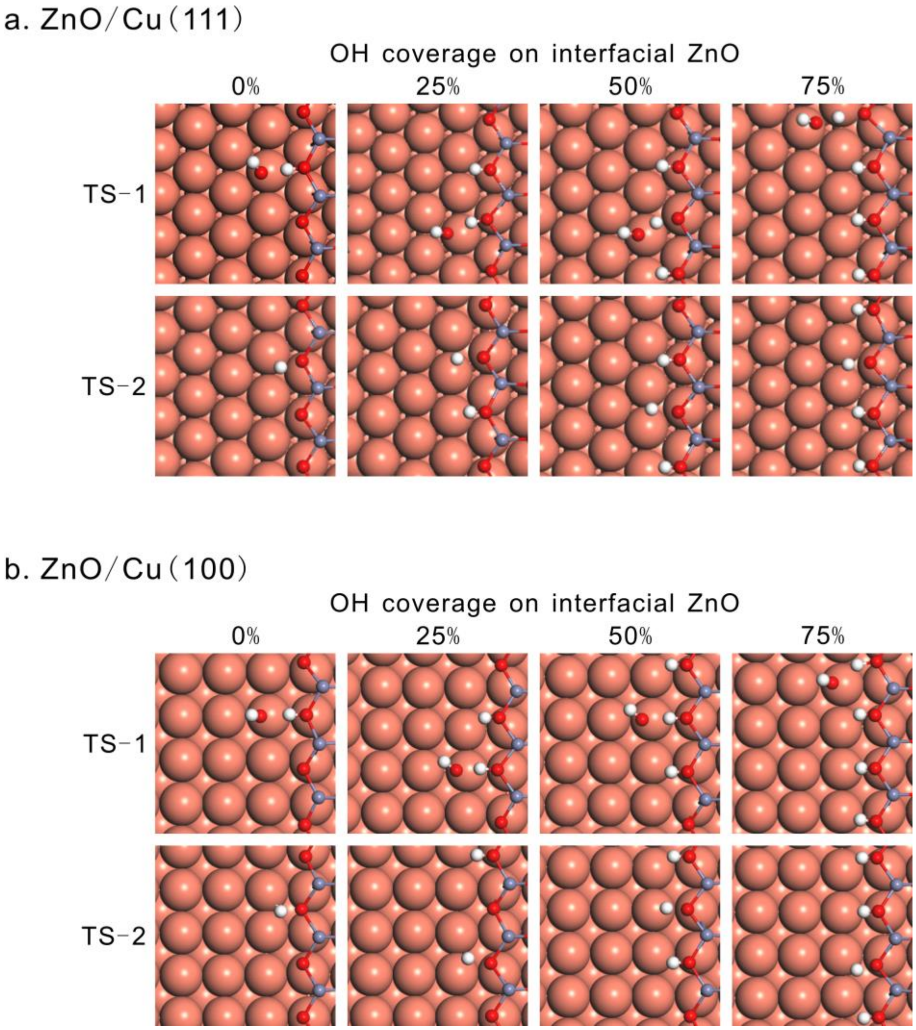

Supplementary Figure 28. The structures at the transition states of H_2O dissociation (TS-1) and H transfer (TS-2) on (**a**) ZnO/Cu(111) and (**b**) ZnO/Cu(100) interfaces with different OH coverages on interfacial ZnO. The reddish-orange, purple, red, grey, and white spheres represent Cu, Zn, O, C, and H atoms, respectively.

Supplementary Table 6. Reaction energy (E_r) and activation energy (E_a) of H_2O dissociation and H transfer on ZnO/Cu(111) and ZnO/Cu(100) interfaces with different OH coverages on interfacial ZnO.

OH coverage on interfacial ZnO	Elementary reactions	ZnO/Cu(111)		ZnO/Cu(100)	
		E_r (eV)	E_a (eV)	E_r (eV)	E_a (eV)
0%	$H_2O_{Cu} \rightarrow OH_{Cu} + O_{ZnO}H$	-0.21	0.22	-0.14	0.15
	$O_{ZnO}H \rightarrow O_{ZnO} + H_{Cu}$	1.00	1.65	0.90	1.39
25%	$H_2O_{Cu} \rightarrow OH_{Cu} + O_{ZnO}H$	-0.11	0.43	-0.13	0.32
	$O_{ZnO}H \rightarrow O_{ZnO} + H_{Cu}$	0.86	1.46	0.76	1.14
50%	$H_2O_{Cu} \rightarrow OH_{Cu} + O_{ZnO}H$	0.04	0.81	-0.02	0.62
	$O_{ZnO}H \rightarrow O_{ZnO} + H_{Cu}$	0.67	1.09	0.64	0.90
75%	$H_2O_{Cu} \rightarrow OH_{Cu} + O_{ZnO}H$	0.38	1.05	0.49	0.87
	$O_{ZnO}H \rightarrow O_{ZnO} + H_{Cu}$	0.45	0.88	0.36	0.76

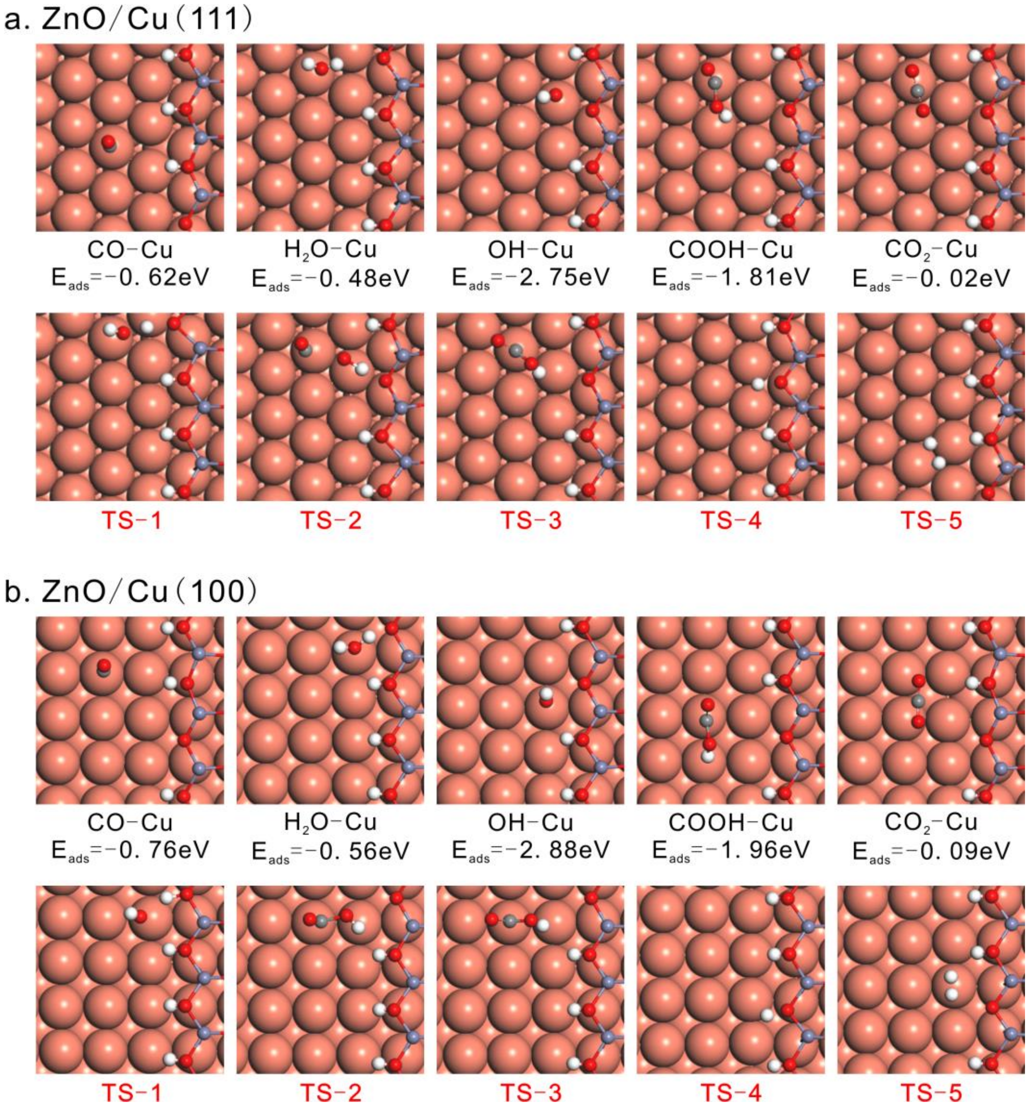

Supplementary Figure 29. The structures with the adsorption energies (E_{ads}) of various adsorption species and the structures at the different transition states of (**a**) ZnO/Cu(111) and (**b**) ZnO/Cu(100) surfaces with 75% OH coverage on interfacial ZnO in catalyzing WGS reaction. The reddish-orange, purple, red, grey, and white spheres represent Cu, Zn, O, C, and H atoms, respectively.

Supplementary Table 7. Reaction energy (E_r) and activation energy (E_a) of each elementary step of WGS reaction on ZnO/Cu(111) and ZnO/Cu(100) surfaces with 75% OH coverage on interfacial ZnO.

Elementary reactions	ZnO/Cu(111)		ZnO/Cu(100)	
	E_r (eV)	E_a (eV)	E_r (eV)	E_a (eV)
$H_2O(g) + Cu_{ZnO\text{-}Cu} \rightarrow H_2O_{Cu}$	-0.48		-0.56	
$CO(g) + Cu_{ZnO\text{-}Cu} \rightarrow CO_{Cu}$	-0.62		-0.76	
$H_2O_{Cu} \rightarrow OH_{Cu} + OH_{ZnO}$	0.38	1.05	0.49	0.87
$CO_{Cu} + OH_{Cu} \rightarrow COOH_{Cu}$	0.10	0.54	0.14	0.45
$COOH_{Cu} \rightarrow CO_{2,Cu} + H_{Cu}$	-0.52	0.72	-0.56	0.68
$CO_{2,Cu} \rightarrow CO_2(g)$	0.02		0.09	
$OH_{ZnO} \rightarrow O_{ZnO} + H_{Cu}$	0.42	0.88	0.36	0.76
$H_{Cu} + H_{Cu} \rightarrow H_2(g)$	0.06	0.83	0.16	0.71

Supplementary Table 8. Catalytic performance of various Cu NCs and ZnO/Cu catalysts towards CO hydrogenation to methanol reaction[a].

Catalysts	CO conversion [%]	Selectivity of CH_3OH+CH_4+C_2^+ [%]		Selectivity of CO_2 [%]
		Total	CH_3OH : CH_4 : C_2^+	
c-Cu-682	3.1	90.4	5.0 : 80.2 : 14.8	9.6
0.5%ZnO/c-Cu-682	3.5	76.8	13.5 : 60.3 : 26.2	23.2
1%ZnO/c-Cu-682	3.9	93.3	23.4 : 66.7 : 9.9	6.7
5%ZnO/c-Cu-682	2.2	73.6	2.0 : 64.3 : 33.7	26.4
o-Cu	1.2	78.4	6.1 : 72.3 : 21.6	21.6
1%ZnO/o-Cu	2.0	78.3	5.9 : 74.3 : 19.8	21.7
5%ZnO/o-Cu	3.2	90.6	0.0 : 85.8 : 14.2	9.4
d-Cu	0	/	/	/
1%ZnO/d-Cu	0	/	/	/
5%ZnO/d-Cu	0	/	/	/
c-Cu-109	3.8	77.9	0.0 : 80.4 : 19.6	22.1
1%ZnO/c-Cu-109	1.6	40.0	0.1 : 75.7 : 24.2	60.0
5%ZnO/c-Cu-109	3.8	73.0	19.8 : 67.2 : 13.0	27.0
9%ZnO/c-Cu-109	2.8	31.2	3.1 : 70.8 : 26.1	68.8
c-Cu-34	9.8	98.1	2.5 : 83.6 : 13.9	1.9
1%ZnO/c-Cu-34	5.4	86.8	0.1 : 85.4 : 14.5	13.2
5%ZnO/c-Cu-34	3.6	85.6	59.1 : 40.0 : 0.9	14.4
9%ZnO/c-Cu-34	7.6	93.4	65.9 : 26.0 : 8.1	6.6
11%ZnO/c-Cu-34	6.0	93.5	0.2 : 80.8 : 19.0	6.5
Cu/ZnO/Al_2O_3[b]	9.4	84.3	90.6 : 8.5 : 0.9	15.7
Cu/ZnO/Al_2O_3[c]	14.5	90.7	17.1 : 27.1 : 55.8	9.3

[a] Reaction conditions: T = 523 K, H_2/CO = 2/1, P = 2 MPa, GHSV = 3600 mL g^{-1} h^{-1}, t = 5 h;

[b] Commercial methanol synthesis catalyst;

[c] Commercial water gas shift catalyst.

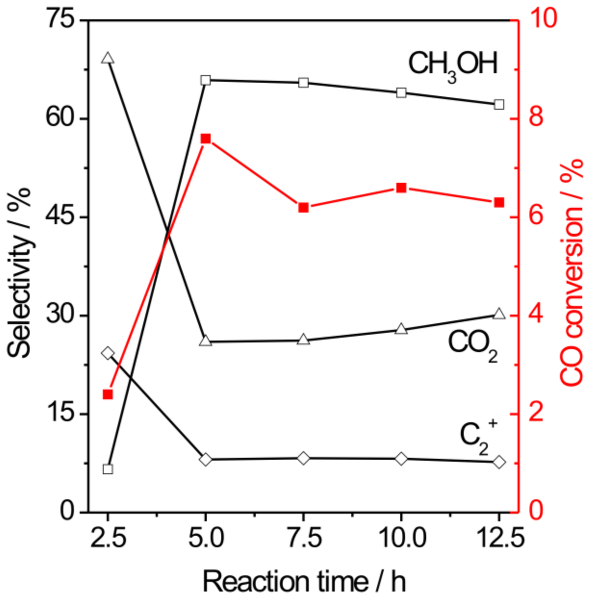

Supplementary Figure 30. The catalytic performance as a function of reaction time over 9%ZnO/c-Cu-34 catalyst in CO hydrogenation to methanol reaction. The results display that the initial CO conversion and CH_3OH selectivity are lower (reaction time = 2.5 h) and reach the maximum at 5 h, subsequently, the catalytic performance slightly decrease. This demonstrates that the restructuring occurs during the CO hydrogenation reaction for 5 h to in situ formed CuZn alloy. The subsequent decrease of catalytic performance is likely due to the sight catalyst deactivation.

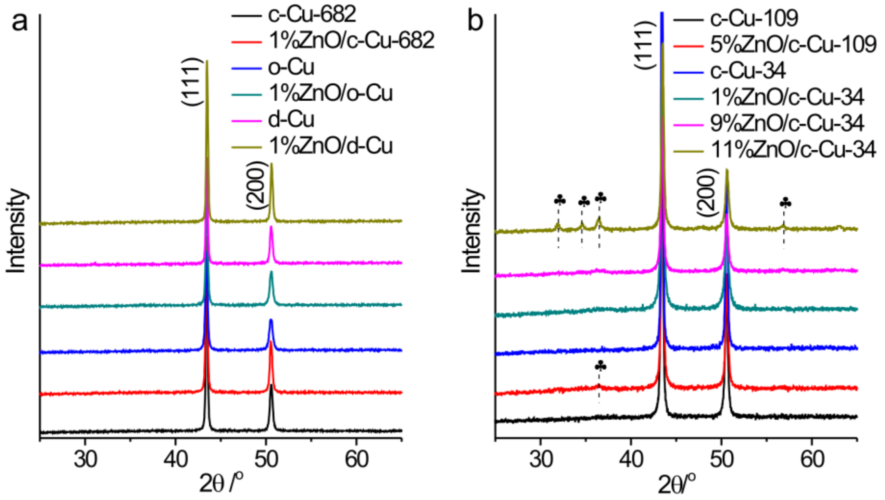

Supplementary Figure 31. (**a**) XRD patterns of various Cu NCs and ZnO/Cu catalysts evaluated after CO hydrogenation to methanol reaction; (**b**) XRD patterns of various c-Cu NCs and ZnO/c-Cu catalysts evaluated after CO hydrogenation to methanol reaction (♣: ZnO). The results exhibit that only Cu crystal phases are observed on representational Cu NCs and ZnO/Cu catalysts, suggesting that ZnO is highly dispersed on these catalysts, except 11%ZnO/c-Cu-34 catalyst, on which ZnO crystal phases prominently exist.

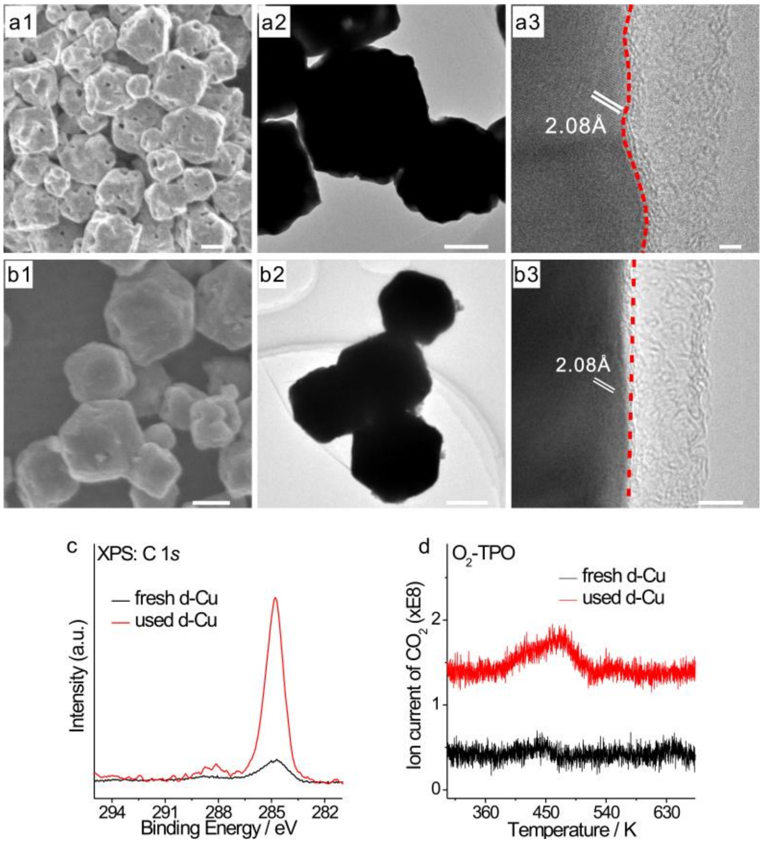

Supplementary Figure 32. The scale bars of (**a1, a2**) and (**b1, b2**) correspond to 500 nm, that of (**b3**) corresponds to 5 nm, and that of (**a3**) corresponds to 2 nm. SEM, TEM, and HRTEM images of (**a1-a3**) d-Cu and (**b1-b3**) 1%ZnO/d-Cu-NC catalysts evaluated after the CO hydrogenation to methanol reaction; (**c**) C 1*s* XPS spectra and (**d**) O_2-TPO profiles of d-Cu NCs and d-Cu NCs evaluated after CO hydrogenation to methanol reaction. Lattice fringe of 2.08 Å corresponds to the spacing of Cu{111} (JCPDS card NO. 89-2838) crystal planes. The surfaces of used d-Cu and 1%ZnO/d-Cu-NC catalysts formed a film with a thickness of about 5-10 nm in the HRTEM images, likely due to amorphous carbon. C 1*s* XPS spectra and O_2-TPO profile of fresh and used d-Cu NCs further confirm that the accumulation of carbonaceous species appears on used d-Cu NCs.

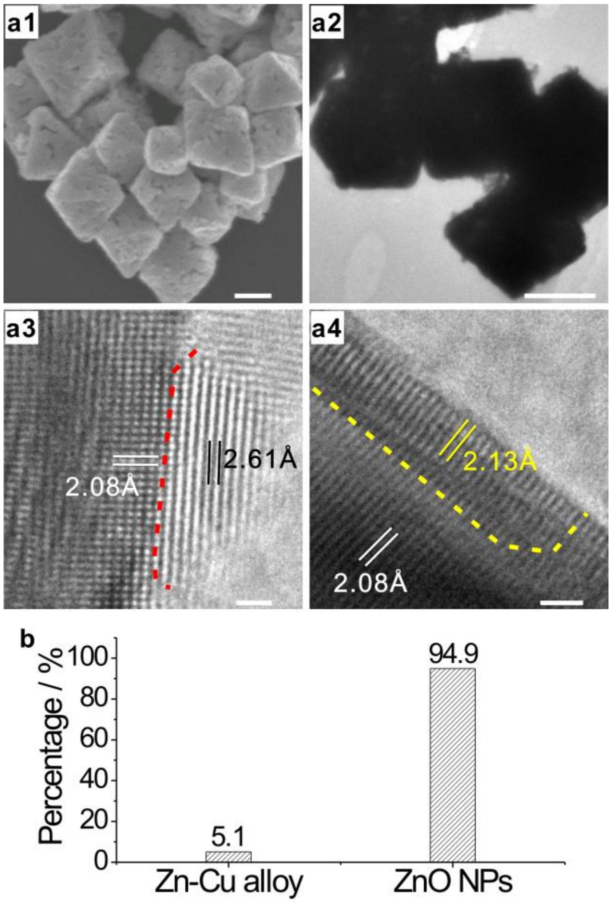

Supplementary Figure 33. The scale bars of (**a1, a2**) correspond to 500 nm and those of (**a3, a4**) correspond to 1 nm. SEM, TEM, and HRTEM images of (**a1-a4**) 1%ZnO/o-Cu NCs evaluated after the CO hydrogenation to methanol reaction; **(b)** Histogram of the percentages of Zn-Cu alloy and ZnO nanoparticles in used 1%ZnO/o-Cu catalyst acquired by counting more than 100 particles in the HRTEM images. Lattice fringes of 2.08, 2.13, and 2.61 Å respectively correspond to the spacing of Cu{111} (JCPDS card NO. 89-2838), Zn-Cu alloy {111}, hexagonal ZnO{002} (JCPDS card NO 89-1397) crystal planes.

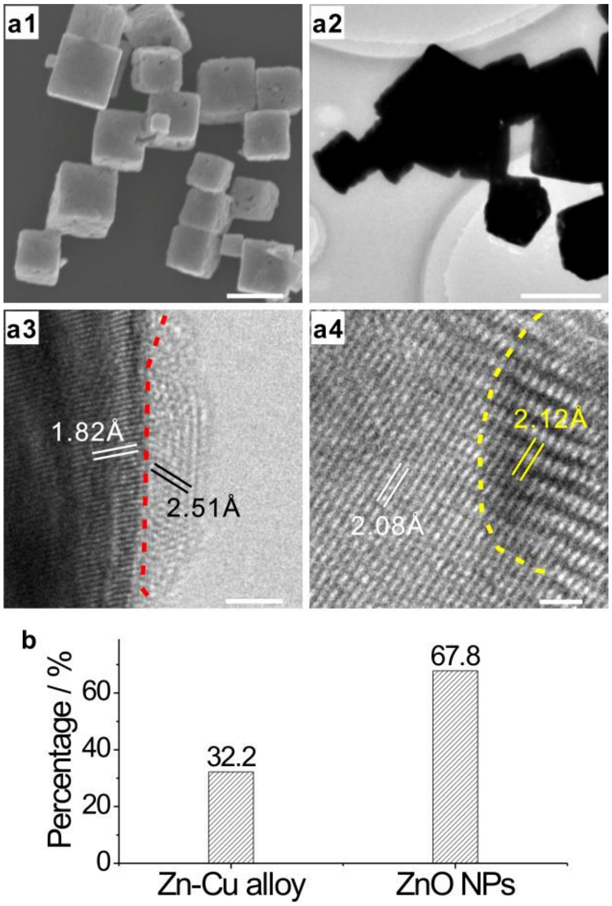

Supplementary Figure 34. The scale bars of (**a1, a2**) correspond to 1 μm, that of (**a3**) corresponds to 2 nm, and that of (**a4**) corresponds to 1 nm. SEM, TEM, and HRTEM images of (**a1-a4**) 1%ZnO/c-Cu-682 evaluated after the CO hydrogenation to methanol reaction; **(b)** Histogram of the percentages of Zn-Cu alloy and ZnO nanoparticles in used 1%ZnO/c-Cu-682 catalyst acquired by counting more than 100 particles in the HRTEM images. Lattice fringes of 1.82, 2.08, 2.12, and 2.51 Å respectively correspond to the spacing of Cu{100}, Cu{111} (JCPDS card NO. 89-2838), Zn-Cu alloy {111}, hexagonal ZnO{101} (JCPDS card NO 89-1397) crystal planes.

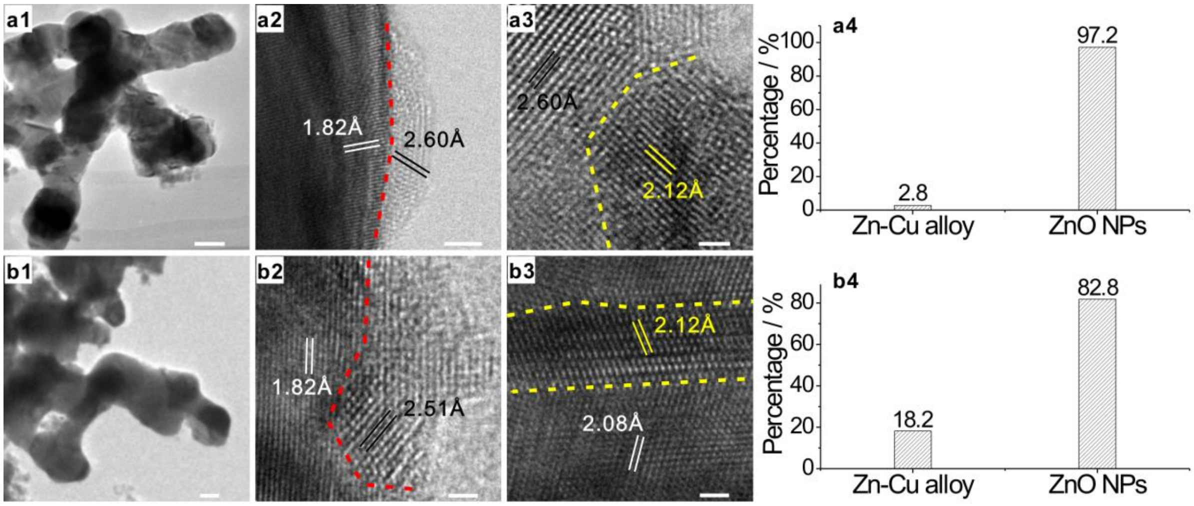

Supplementary Figure 35. The scale bars of (**a1**) and (**b1**) correspond to 50 nm, that of (**a2**) corresponds to 2 nm, and those of (**a3**) and (**b2, b3**) correspond to 1 nm. TEM and HRTEM images of (**a1-a3**) 1%ZnO/c-Cu-109 and (**b1-b3**) 5%ZnO/c-Cu-109 catalysts evaluated after the CO hydrogenation to methanol reaction; Histogram of the percentages of Zn-Cu alloy and ZnO nanoparticles in used (**a4**) 1%ZnO/c-Cu-109 and (**b4**) 5%ZnO/c-Cu-109 catalysts acquired by counting more than 100 particles in the HRTEM images. Lattice fringes of 1.82, 2.08, 2.12, 2.51, and 2.60 Å respectively correspond to the spacing of Cu{100}, Cu{111} (JCPDS card NO. 89-2838), Zn-Cu alloy {111}, ZnO{101}, and ZnO{002} (JCPDS card NO 89-1397) crystal planes.

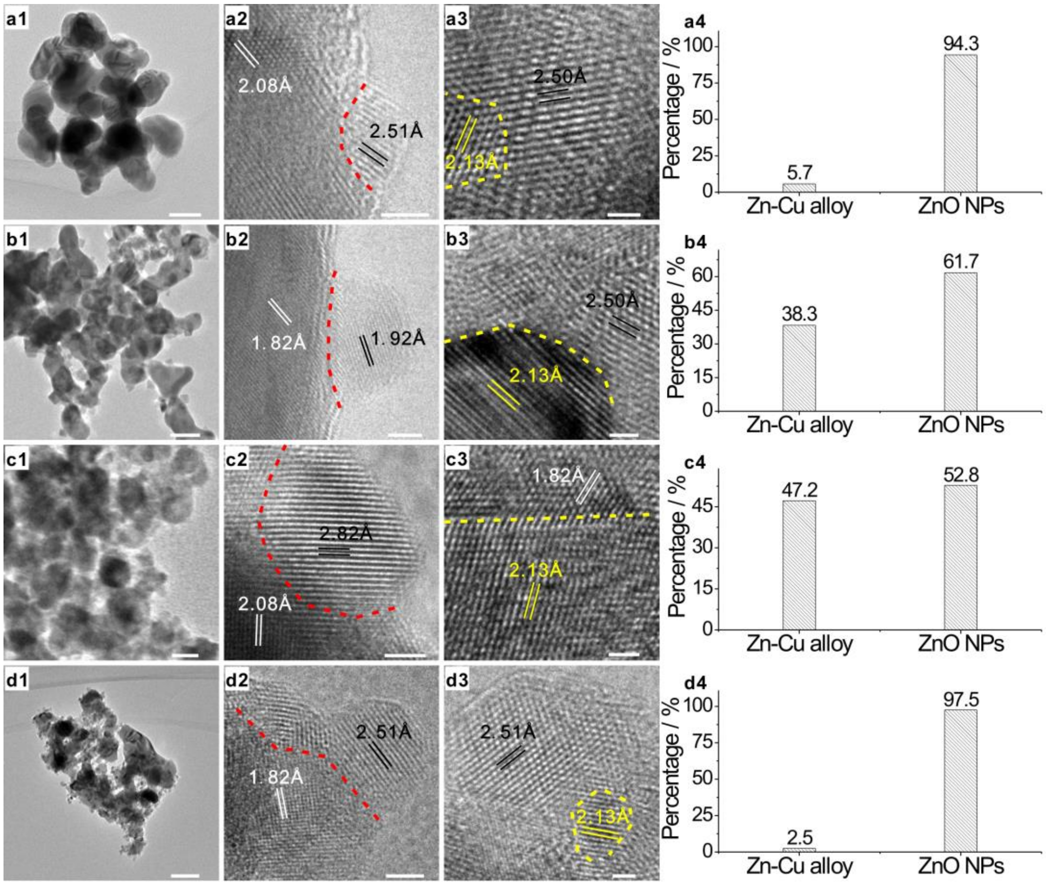

Supplementary Figure 36. The scale bars of (**a1**), (**b1**) and (**d1**) correspond to 50 nm, that of (**c1**) corresponds to 20 nm, those of (**a2-d2**) correspond to 2 nm, and those of (**a3-d3**) correspond to 1 nm. SEM, TEM, and HRTEM images of (**a1-a3**) 1%ZnO/c-Cu-34, (**b1-b3**) 5%ZnO/c-Cu-34, (**c1-c3**) 9%ZnO/c-Cu-34, and (**d1-d3**) 11%ZnO/c-Cu-34 evaluated after the CO hydrogenation to methanol reaction; Histogram of the percentages of Zn-Cu alloy and ZnO nanoparticles in used (**a4**) 1%ZnO/c-Cu-34, (**b4**) 5%ZnO/c-Cu-34, (**c4**) 9%ZnO/c-Cu-34, and (**d4**) 11%ZnO/c-Cu-34 acquired by counting more than 100 particles in the HRTEM images. Lattice fringes of 1.82, 2.08, 2.13, 1.92, 2.50-2.51, and 2.82 Å respectively correspond to the spacing of Cu{100}, Cu{111} (JCPDS card NO. 89-2838), Zn-Cu alloy {111}, ZnO{102}, ZnO{101}, and ZnO{100} (JCPDS card NO 89-1397) crystal planes.

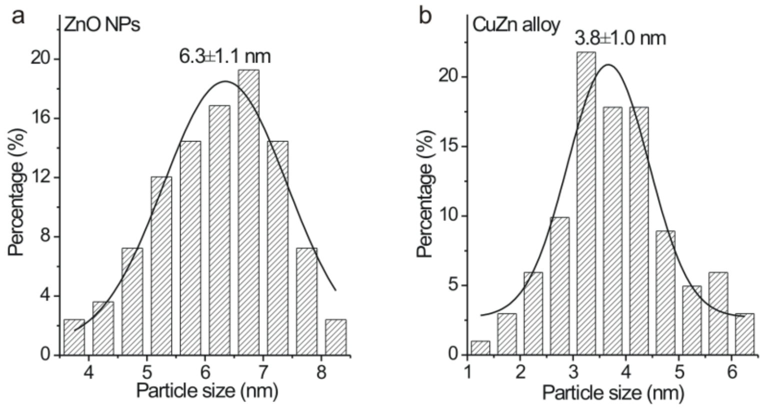

Supplementary Figure 37. The size distributions of (a) ZnO particles in fresh 9%ZnO/c-Cu-34 catalyst and (b) CuZn alloy in used 9%ZnO/c-Cu-34 catalyst.

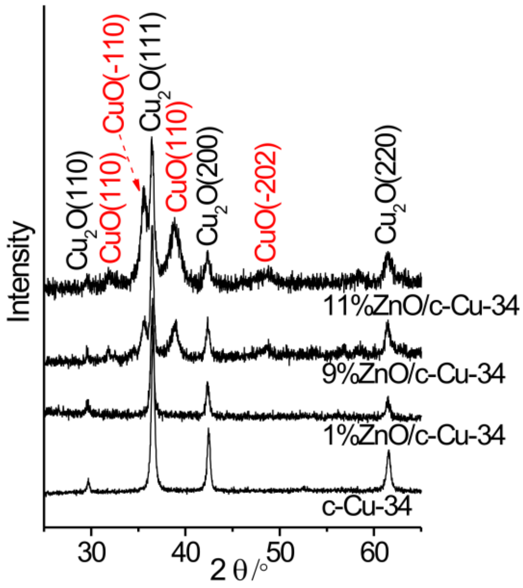

Supplementary Figure 38. XRD patterns of c-Cu-34 and various ZnO/c-Cu-34 catalysts evaluated after CO hydrogenation reaction. The presence of Cu_2O and CuO in used catalysts can be ascribed the oxidation of used catalysts exposed to air.

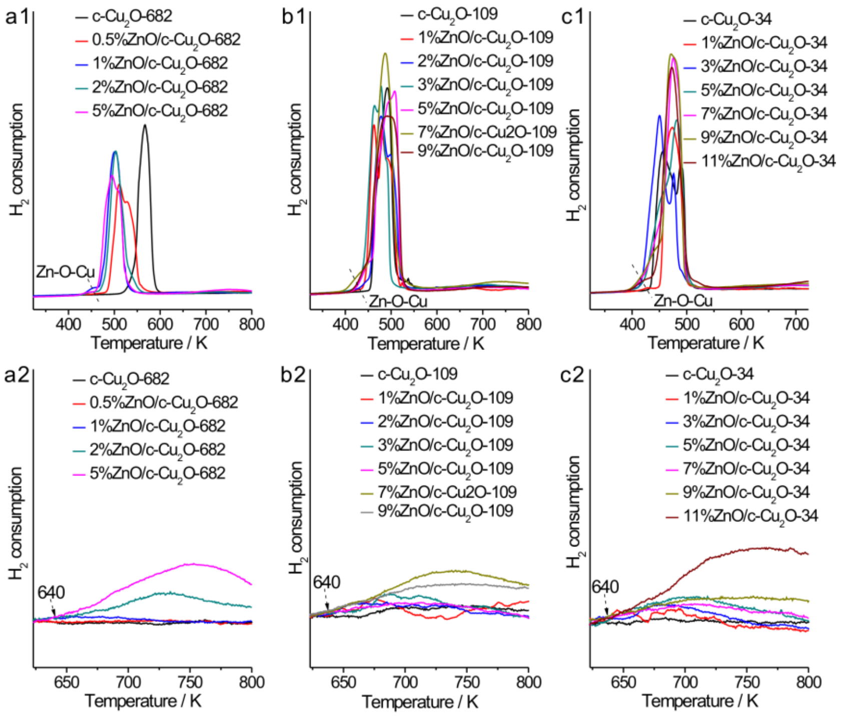

Supplementary Figure 39. H_2-TPR profiles of (**a1, a2**) c-Cu_2O-682 NCs and various ZnO/c-Cu_2O-682 catalysts, (**b1, b2**) c-Cu_2O-109 NCs and various ZnO/c-Cu_2O-109 catalysts, and (**c1, c2**) c-Cu_2O-34 NCs and various ZnO/c-Cu_2O-34 catalysts. Supplementary Fig. 38 A2-C2 is the enlarged pictures of corresponding Supplementary Fig. 38 A1-C1. According to the H_2-TPR results, the profiles are composed of the reductions of three components, respectively are ZnO-Cu_xO interface (denoted as Zn-O-Cu), CuO&Cu_2O, and large ZnO particles [26-28]. For ZnO/c-Cu_2O-682 catalysts, it is obvious to observe that the reduction strength of Zn-O-Cu structure increases with ZnO loading and reaches the maximum for 1%ZnO/c-Cu_2O-682 catalyst, and then decreases, indicating that ZnO with 1% wt. supported on c-Cu_2O-682 occupies the most number of Zn-O-Cu structure. However, such clear conclusion is difficult to be observed for ZnO/c-Cu_2O-109 and ZnO/c-Cu_2O-34 catalysts due to the partial overlap of the reduction of Zn-O-Cu structure with the reductions of CuO&Cu_2O. In addition, the reduction of ZnO particles, starting from 640 K, is present for ZnO/Cu_2O catalysts with high ZnO loadings, suggesting large ZnO particles not in favor for the formation of CuZn alloy.

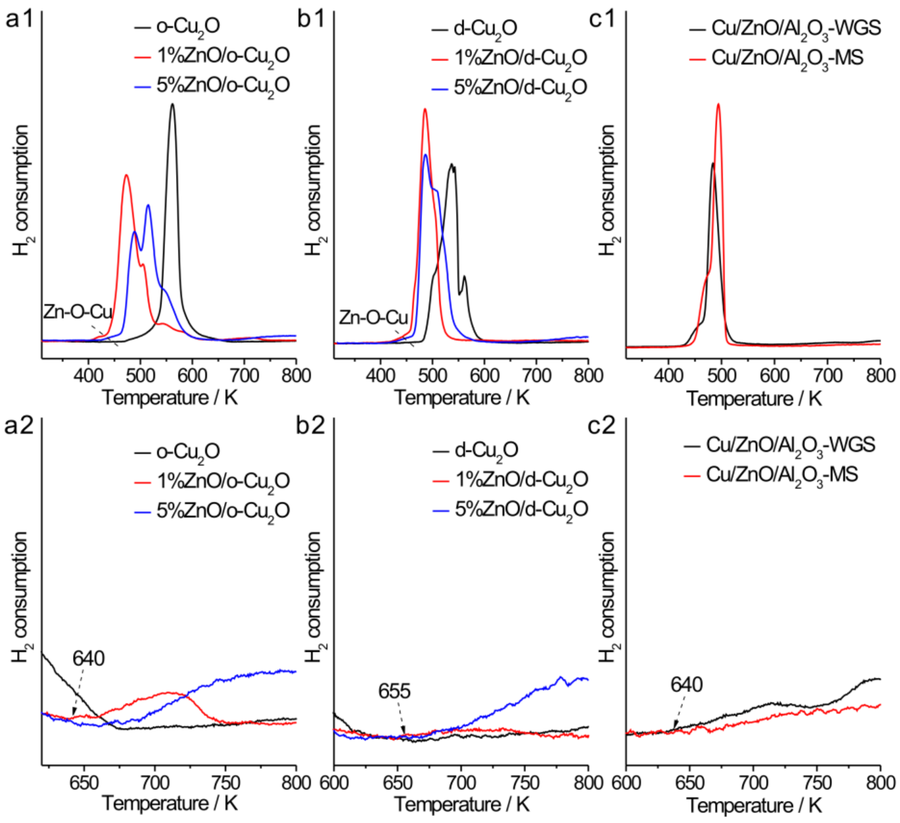

Supplementary Figure 40. H_2-TPR profiles of (**a1, a2**) o-Cu_2O NCs and various ZnO/o-Cu_2O catalysts, (**b1, b2**) d-Cu_2O NCs and various ZnO/d-Cu_2O catalysts, and (**c1, c2**) commercial water-gas shift and CO hydrogenation to methanol catalysts. Supplementary Fig. 39 A2-C2 are the enlarged pictures of corresponding Supplementary Fig. 39 A1-C1. The results are similar to those of ZnO/c-Cu_2O catalysts introduced in the caption of Supplementary Fig. 38.

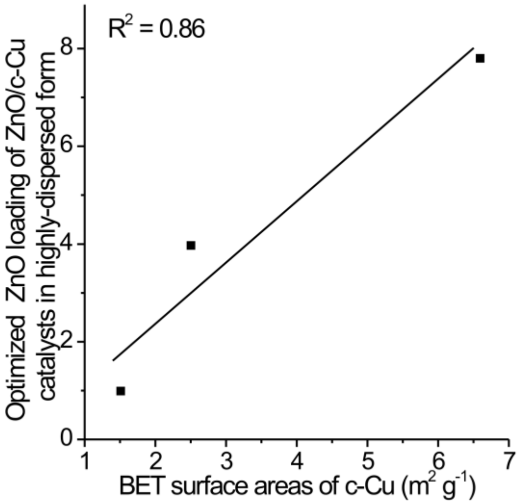

Supplementary Figure 41. The optimized ZnO loading of ZnO/c-Cu catalysts in highly-dispersed form as a function of the BET surface areas of c-Cu NCs. The maximum ZnO loading of ZnO/c-Cu catalysts in highly-dispersed form was derived based on the H_2-TPR results in Supplementary Fig. 39, which is 0.99, 3.97, and 7.80 wt.% for ZnO/c-Cu-682, ZnO/c-Cu109, and ZnO/c-Cu-34, respectively.

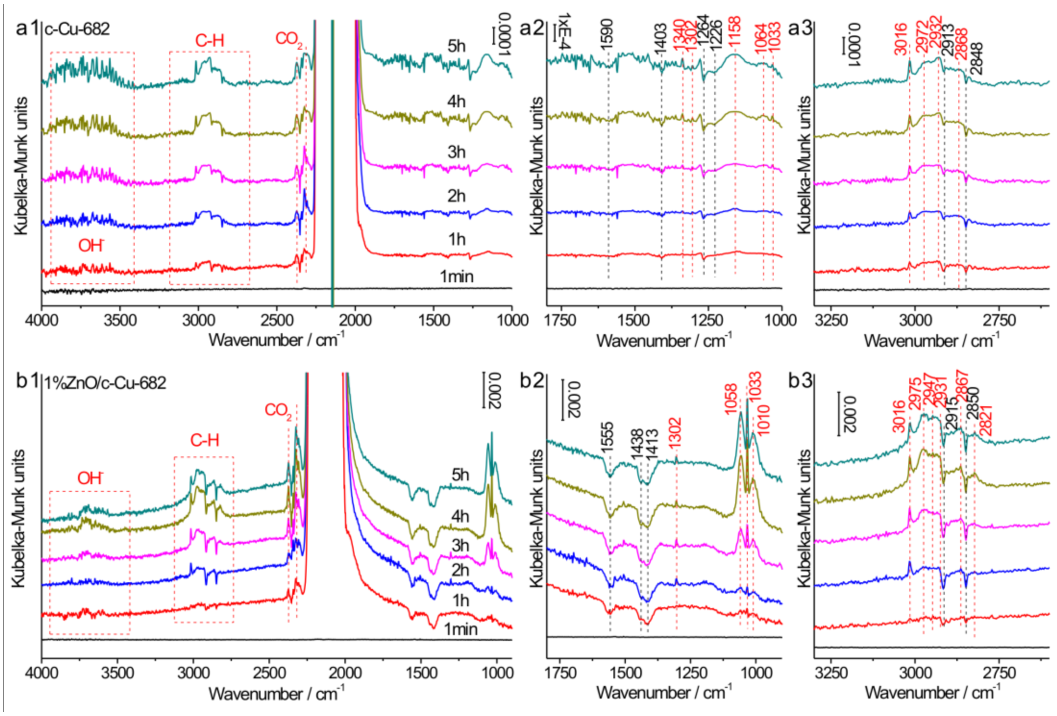

Supplementary Figure 42. In-situ DRIFTS of 2 MPa 33.3% CO + 66.7% H_2 on (**a1-a3**) c-Cu-682 and (**b1-b3**) 1%ZnO/c-Cu-682 catalysts at 523 K.

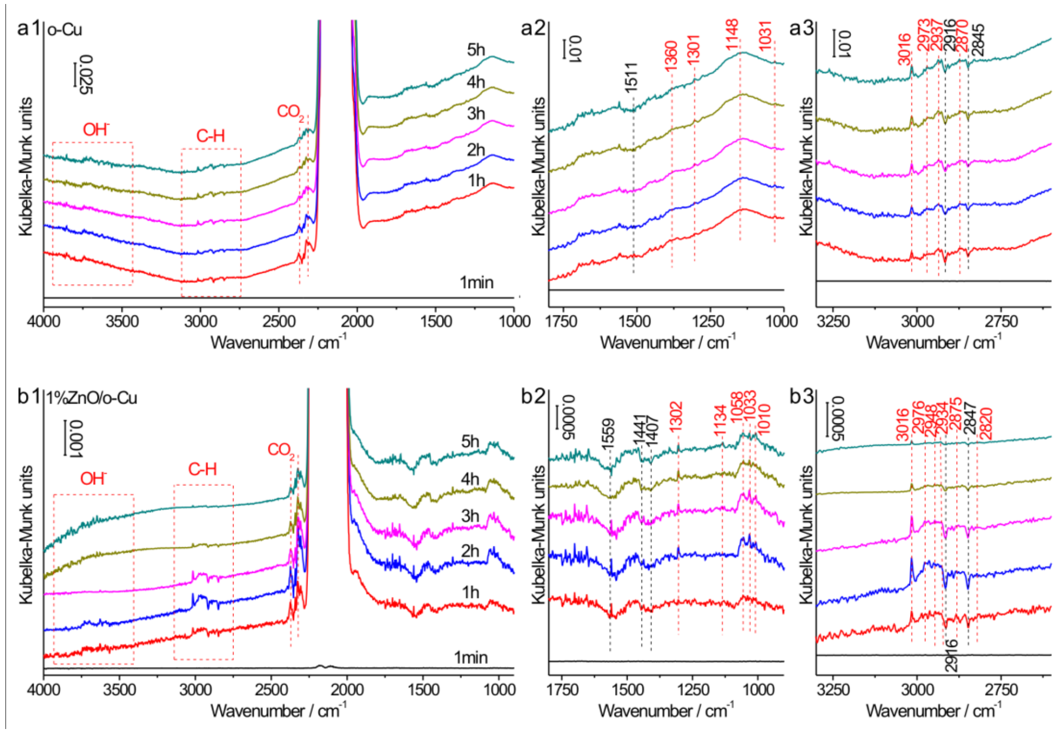

Supplementary Figure 43. In-situ DRIFTS of 2 MPa 33.3% CO + 66.7% H_2 on (**a1-a3**) o-Cu and (**b1-b3**) 1%ZnO/o-Cu catalysts at 523 K.

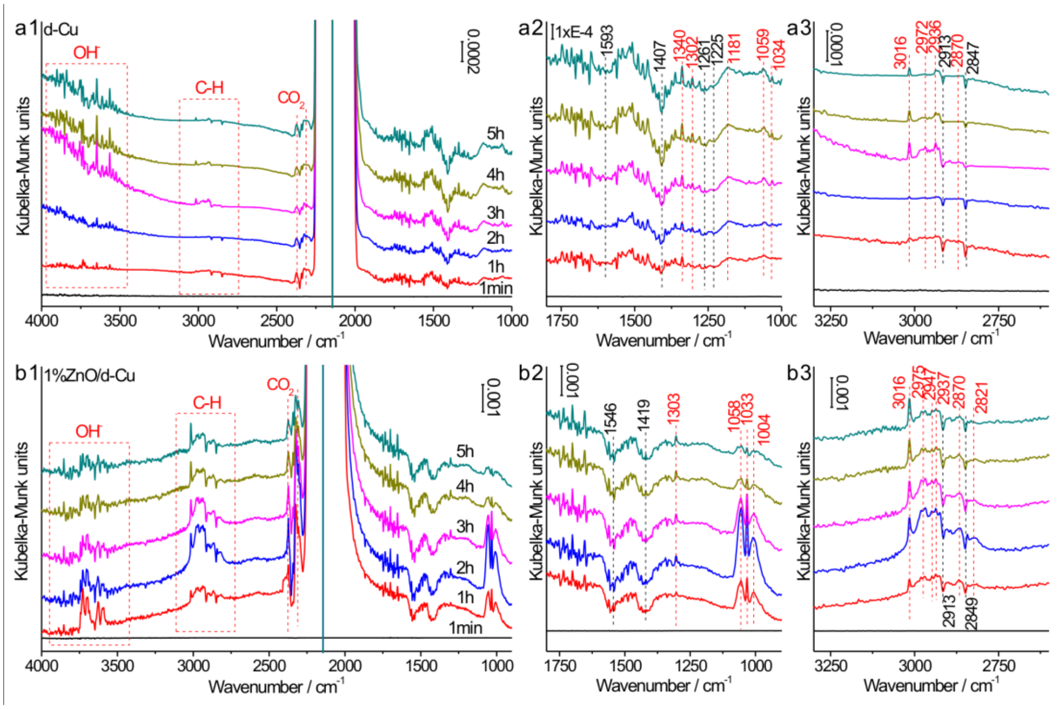

Supplementary Figure 44. In-situ DRIFTS of 2 MPa 33.3% CO + 66.7% H_2 on (**a1-a3**) d-Cu and (**b1-b3**) 1%ZnO/d-Cu catalysts at 523 K.

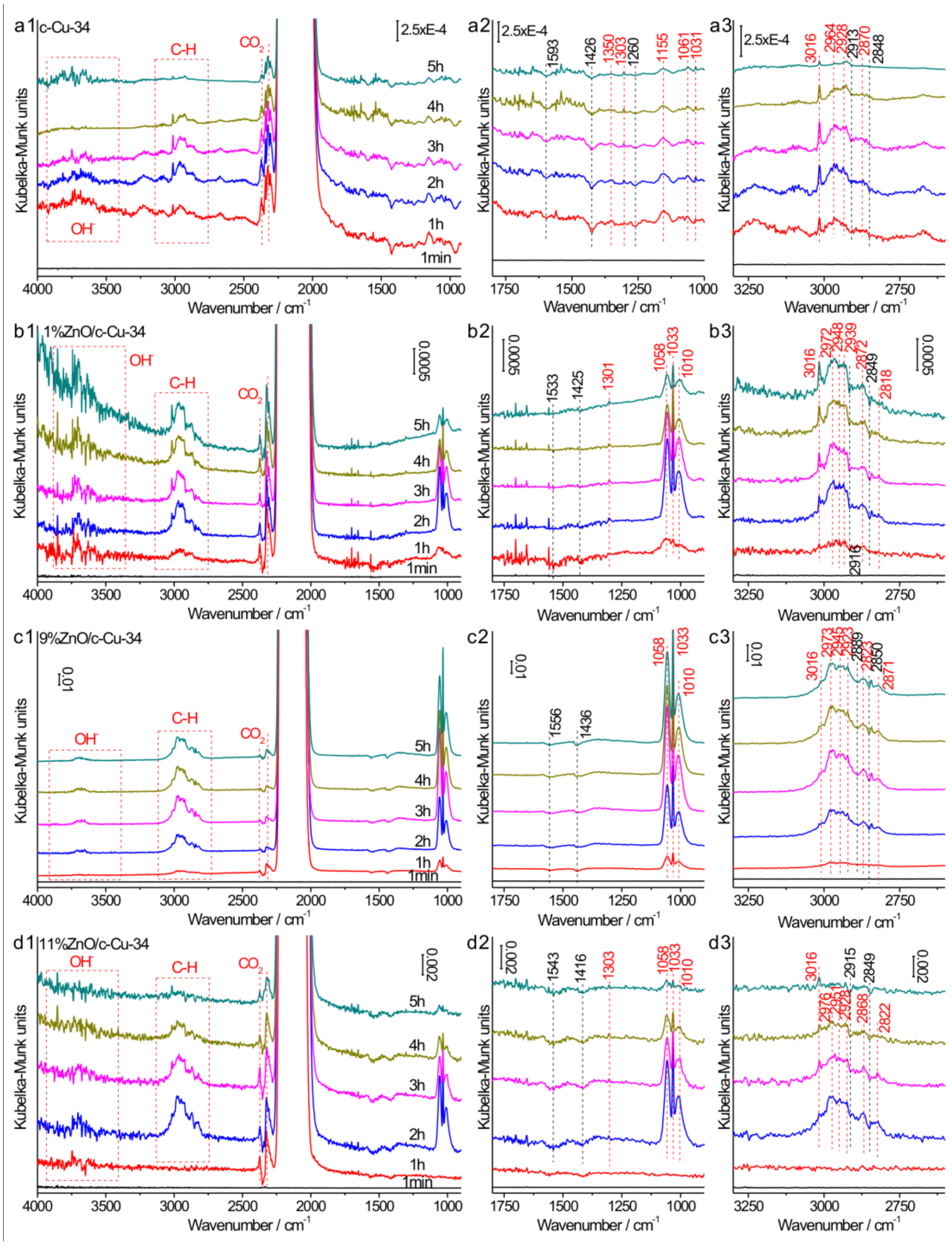

Supplementary Figure 45. In-situ DRIFTS of 2 MPa 33.3% CO + 66.7% H_2 on (**a1-a3**) c-Cu-34, (**b1-b3**) 1%ZnO/c-Cu-34, (**c1-c3**) 9%ZnO/c-Cu-34, and (**d1-d3**) 11%ZnO/c-Cu-34 catalysts at 523 K.

Supplementary Table 9. Assignment of vibrational bands in the in situ DRIFTS spectra of surface species at various Cu and ZnO/Cu catalysts.

Assignments	Bands (cm^{-1})						Ref.
	c-Cu	ZnO/c-Cu	o-Cu	ZnO/o-Cu	d-Cu	ZnO/d-Cu	
CH_3O_a	1061~1064	1010<br>1058<br>2818~2823<br>2947~2951	-	1010<br>1058<br>2820<br>2948	1059	1004<br>1058<br>2821<br>2947	[28-30]
$CH_3OH(g)$	1031~1033	1033<br>2818~2823<br>2947~2951	1031	1033<br>2820<br>2948	1034	1033<br>2821<br>2947	[31]
CH_2OH_a	1155~1158<br>1340~1350	-	1148<br>1360	1134	1181<br>1340	-	[32, 33]
$CH_{2,a}$	2868~2870<br>2928~2932	2867~2872<br>2923~2939	2870<br>2937	2875<br>2934	2870<br>2936	2870<br>2937	[34, 35]
$CH_{3,a}$	2964~2972	2972~2976	2973	2976	2972	2975	[34, 35]
$CH_4(g)$	1302~1303<br>3016	1301~1303<br>3016	1301<br>3016	1302<br>3016	1302<br>3016	1303<br>3016	[35, 36]
Formate	1590~1593<br>2848<br>2913	1533~1556<br>2848~2850<br>2889~2915	2845<br>2916	1559<br>2847<br>2916	1593<br>2847<br>2913	1546<br>2849<br>2913	[17, 37, 38]
Carbonates	1226<br>1260~1264<br>1403~1426	1413~1416<br>1425~1438	1511	1407<br>1441	1225<br>1261<br>1407	1419	[29, 38-40]
Hydroxyl groups	3500~3700						[41]

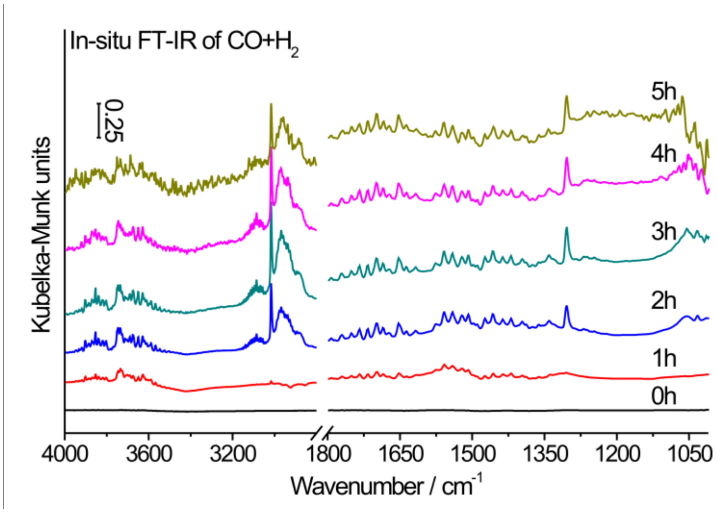

Supplementary Figure 46. In-situ FT-IR of 2 MPa 33.3% CO + 66.7% H_2 on 1%ZnO/c-Cu-682 at 523 K.

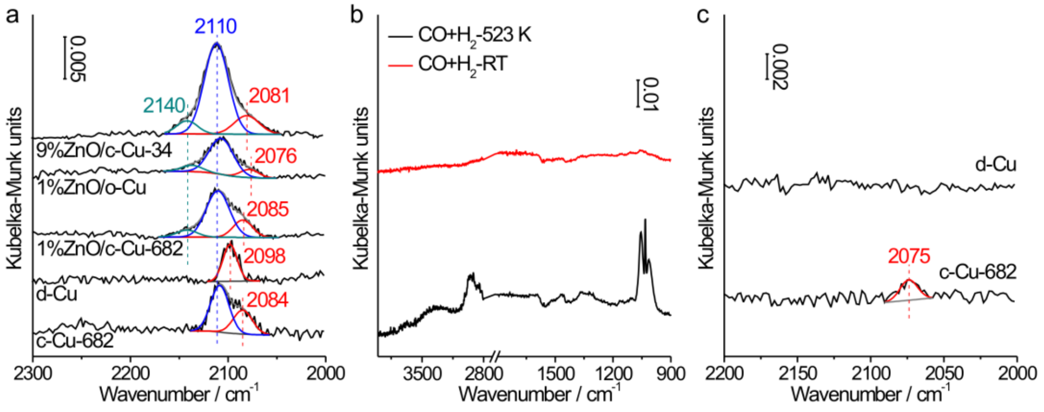

Supplementary Figure 47. (a) In situ FT-IR results of CO adsorbed on various fresh Cu and ZnO/Cu catalysts acquired by the reduction of 5% H_2/Ar at 523 K for 2 h. The red, blue, and light blue lines represent the fitted vibrational peaks of CO adsorbed on the Cu facets, defective sites of Cu, and Cu-ZnO interface, respectively; **(b)** In situ DRIFTS results of 2 MPa 33.3% CO + 66.7% H_2 on 9%ZnO/c-Cu-34 at 548 K and then low down to room temperature; **(c)** In situ FT-IR results of CO adsorbed on various used Cu NCs. According to the results in Supplementary Fig. 46A, it proves that the surface structures are similar whether various Cu_2O and ZnO/Cu_2O catalysts is reduced by 5% CO/Ar or 5% H_2/Ar, demonstrating that the initial structures of various Cu and ZnO/Cu catalysts used for WGS and CO hydrogenation to methanol reactions are close. The results in Supplementary Fig. 46B demonstrate that the surface species formed on reaction atmosphere are facilely desorbed, thus it is no effect on the in situ FT-IR results of CO adsorbed on used catalyst surfaces.

Supplementary Table 10. The fitting parameters of in situ FT-IR of CO adsorption on various used ZnO/Cu catalysts after CO hydrogenation reaction.

Catalysts	~2060 cm^{-1}			~2075 cm^{-1}		
	FWHM /cm^{-1}	L-G %	Areas	FWHM /cm^{-1}	L-G %	Areas
1%ZnO/o-Cu	20	10	0.026	16	10	0.022
0.5%ZnO/c-Cu-682	16	10	0.011	18	10	0.018
1%ZnO/c-Cu-682	16	10	0.021	15	10	0.017
5%ZnO/c-Cu-109	20	10	0.023	20	10	0.028
5%ZnO/c-Cu-34	20	10	0.036	19	10	0.030
9%ZnO/c-Cu-34	15	10	0.09	18	10	0.010

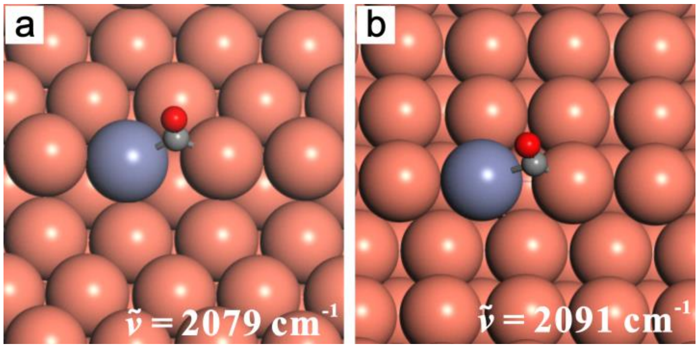

Supplementary Figure 48. Optimized structures of stable CO species adsorbed on (**a**) ZnCu(611) and (**b**) ZnCu(211) surfaces with the C=O stretching frequencies. Red, gray, and pink balls represent O, C, and Cu atoms, respectively. The vibrational frequency of CO adsorbed on ZnCu(611) and ZnCu(211) surfaces is about 30 and 10 cm^{-1} lower than that on Cu(100) and Cu(111) surfaces, respectively, which are consistent with the experimental observations.

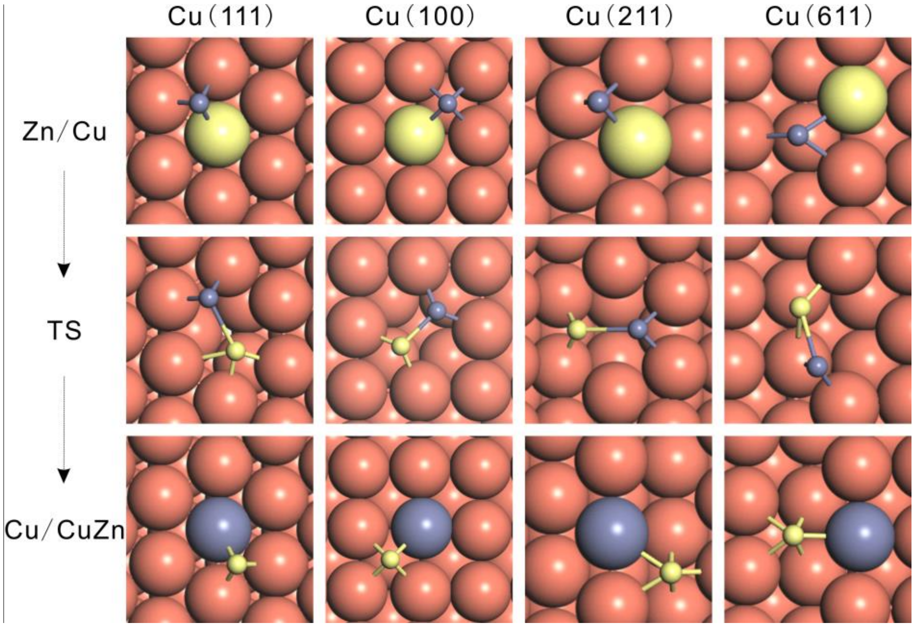

Supplementary Figure 49. The structures of the formation process of Cu-Zn alloy over various Cu surfaces. The reddish-orange, yellow, and purple spheres represent Cu, replaced Cu by Zn, and Zn atoms, respectively.

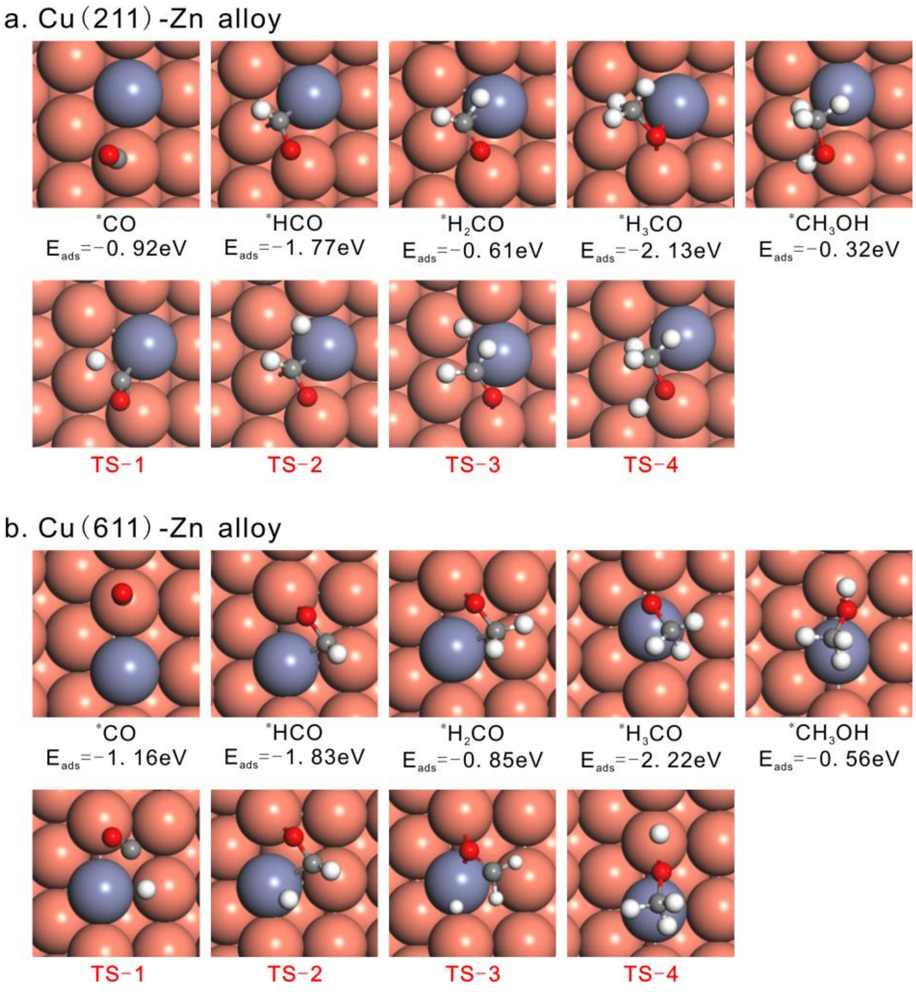

Supplementary Figure 50. The structures with the adsorption energies (E_{ads}) of various adsorption species and the structures at the different transition states of **(a)** Cu(211)-Zn and **(b)** Cu(611)-Zn alloys in catalyzing CO hydrogenation to methanol. The reddish-orange, purple, red, grey, and white spheres represent Cu, Zn, O, C, and H atoms, respectively.

Supplementary Table 11. Reaction energy (E_r) and activation energy (E_a) of each elementary step of CO hydrogenation to methanol reaction on Cu(211)-Zn and Cu(611) alloys.

Elementary Reactions	Cu(211)-Zn alloy		Cu(611)-Zn alloy	
	E_r (eV)	E_a (eV)	E_r (eV)	E_a (eV)
CO(g) + * → *CO	-0.92		-1.16	
*CO + *H →*HCO	0.42	0.76	0.40	0.69
*HCO + *H →*H_2CO	-0.25	0.69	-0.34	0.66
*H_2CO + *H → *H_3CO	-1.14	0.31	-0.98	0.19
*H_3CO + *H → *CH_3OH	0.21	1.02	0.16	0.85
*CH_3OH → CH_3OH(g) + *	0.32		0.56	

Supplementary References

1. Kresse, G., Joubert, D. From ultrasoft pseudopotentials to the projector augmentedwave method. *Phys. Rev. B* **59**, 1758-1775 (1999).

2. Kresse, G., Furthmuller, J. Efficient iterative schemes for ab initio total-energy calculations using a plane-wave basis set. *Phys. Rev. B* **54**, 11169-11186 (1996).

3. Blochl, P. E. Projector augmented-wave method. *Phys. Rev. B* **50**, 17953-17979 (1994).

4. Perdew, J. P., Wang, Y. Pair-distribution function and its coupling-constant average for the spin-polarized electron gas. *Phys. Rev. B* **45**, 12947-12954 (1992).

5. Perdew, J. P., Burke, K., Ernzerhof, M. Generalized gradient approximation made simple. *Phys. Rev. Lett.* **77**, 3865-3868 (1996).

6. Behrens, M. *et al*. The active site of methanol synthesis over Cu/ZnO/Al_2O_3 industrial catalysts. *Science* **336**, 893-897 (2012).

7. Studt, F., Abild-Pedersen, F., Varley, J. B., Nørskov, J. K. CO and CO_2 hydrogenation to methanol calculated using the Beef-Vdw Functional. *Catal. Lett.* **143**, 71-73 (2013).

8. Lunkenbein, T., Schumann, J., Behrens, M., Schloegl, R., Willinger, M. G. Formation of a ZnO overlayer in industrial Cu/ZnO/Al_2O_3 catalysts induced by strong metal-support interactions. *Angew. Chem. Int. Ed.* **54**, 4544-4548 (2015).

9. Deng, X., Yao, K., Sun, K., Li, W.-X., Lee, J., Matranga, C. Growth of single- and bilayer ZnO on Au(111) and interaction with copper. *J. Phys. Chem. C* **117**, 11211-11218 (2013).

10. Schott, V. *et al*. Chemical activity of thin oxide layers: strong interactions with the support yield a new thin-film phase of ZnO. *Angew. Chem. Int. Ed.* **52**, 11925-11929 (2013).

11. Tusche, C., Meyerheim, H. L., Kirschner, J. Observation of depolarized ZnO(0001) monolayers: formation of unreconstructed planar sheets. *Phys. Rev. Lett.* **99**, 026102-026105 (2007).

12. Weirum, G. *et al*. Growth and surface structure of zinc oxide layers on a Pd(111) surface. *J. Phys. Chem. C* **114**, 15432-15439 (2010).

13. Sun, K. J., Zhao, Y. H., Su, H.-Y., Li, W.-X. Force reversed method for locating transition states. *Theor. Chem. Acc.* **131**, 1118-1127 (2012).

14. Henkelman, G., Jonsson, H. Improved tangent estimate in the nudged elastic band method for finding minimum energy paths and saddle points. *J. Chem. Phys.* **113**, 9978-9985 (2000).

15. Henkelman, G., Uberuaga, B. P., Jonsson, H. A Climbing image nudged elastic band method for finding saddle points and minimum energy paths. *J. Chem. Phys.* **113**, 9901-9904 (2000).

16. Janotti, A., Van de Walle, C. G. Native point defects in ZnO. *Phys. Rev. B* **76**, 165202-165223 (2007).

17. Zhang, Z. *et al*. The most active Cu facet for low-temperature water gas shift reaction. *Nat. Commun.* **8**, 488 (2017).

18. Zhang, Z. *et al*. Site-resolved Cu_2O catalysis in the oxidation of CO. *Angew. Chem. Int. Ed*. **58**, 4276-4280 (2019).

19. Chen, S., Cao, T., Gao, Y., Li, D., Xiong, F., Huang, W. Probing surface structures of CeO_2, TiO_2, and Cu_2O nanocrystals with CO and CO_2 chemisorption. *J. Phys. Chem. C* **120**, 21472-21485 (2016).

20. Wadayama, T., Kubo, K., Yamashita, T., Tanabe, T., Hatta, A. Infrared reflection absorption study of carbon monoxide adsorbed on submonolayer Fe-covered Cu (100), (110), and (111) bimetallic surfaces. *J. Phys. Chem. B* **107**, 3768-3773 (2003).

21. Horn, K., Pritchard, J. Infrared spectrum of CO chemisorbed on Cu (100). *Surf. Sci.* **55**, 701-704 (1976).

22. Dulaurent, O., Courtois, X., Perrichon, V., Bianchi, D. Heats of adsorption of CO on a $CuAl_2O_3$ catalyst using FTIR spectroscopy at high temperatures and under adsorption equilibrium conditions. *J. Phys. Chem. B* **104**, 6001-6011 (2000).

23. Hayden, B. E., Kretzschmar, K., Bradshaw, A. M. An infrared spectroscopic study of CO on Cu (111): the linear, bridging and physisorbed species. *Surf. Sci.* **155**, 553-566 (1985)

24. Horn, K., Hussain, M., Pritchard, J. The adsorption of CO on Cu (110). *Surf. Sci.* **63**, 244-253 (1977).

25. Hagman, B. *et al.* Steps control the dissociation of CO_2 on Cu(100). *J. Am. Chem. Soc.* **140**, 12974-12979 (2018).

26. Bao, H. *et al*. Shape-dependent reducibility of cuprous oxide nanocrystals. *J. Phys. Chem. C* **114**, 6676-6680 (2010).

27. Liang, M., Kang, W., Xie, K. Comparison of reduction behavior of Fe_2O_3, ZnO and $ZnFe_2O_4$ by TPR technique. *J. Nat. Gas Chem.* **18**, 110-113 (2009).

28. Wang, J. *et al*. A highly selective and stable ZnO-ZrO_2 solid solution catalyst for CO_2 hydrogenation to methanol. *Sci. Adv.* **3**, 1701290 (2017).

29. Lin, S. D., Cheng, H., Hsiao, T. C. In situ DRIFTS study on the methanol oxidation by lattice oxygen over Cu/ZnO catalyst. *J. Mole. Catal. A: Chem.* **342-343**, 35-40 (2011).

30. Sambeth, J. E., Centeno, M. A., Paúl, A., Briand, L. E., Thomas, H. J., Odriozola, J. A. In situ DRIFTS study of the adsorption-oxidation of CH_3OH on V_2O_5. *J. Mole. Catal. A: Chem.* **161**, 89-97 (2000).

31. Bailey, S. A DRIFTS study of the morphology and surface adsorbate composition of an operating methanol synthesis catalyst. *Catal. Lett.* **20**, 99-111 (1995).

32. Li, H. *et al*. Synergetic interaction between neighbouring platinum monomers in CO_2 hydrogenation. *Nat. Nanotechnol.* **13**, 411-417 (2018).

33. Wu, J. C. S., Huang, C.-W. In situ DRIFTS study of photocatalytic CO_2 reduction under UV irradiation. *Front. Chem. Eng. China* **4**, 120-126 (2010).

34. Panagiotopoulou, P., Kondarides, D. I., Verykios, X. E. Mechanistic aspects of the selective methanation of CO over Ru/TiO_2 catalyst. *Catal. Today* **181**, 138-147 (2012).

35. Eckle, S., Anfang, H.-G., Behm, R. J. Reaction intermediates and side products in the methanation of CO and CO_2 over supported Ru catalysts in H_2-rich reformate gases. *J. Phys. Chem. C* **115**, 1361-1367 (2011).

36. Walter, K., Buyevskaya, O. V., Wolf, D., Baerns, M. Rhodium-catalyzed partial oxidation of methane to CO and H_2. In situ DRIFTS studies on surface intermediates. *Catal. Lett.* **29**, 261-270 (1994).

37. Weigel, J., Koeppel, R. A., Baiker, A., Wokaun, A. Surface species in CO and CO_2 hydrogenation over copper/zirconia: on the methanol synthesis mechanism. *Langmuir* **12**, 5319-5329 (1996).

38. Graciani, J. *et al*. Highly active copper-ceria and copper-ceria-titania catalysts for methanol synthesis from CO_2. *Science* **345**, 546-550 (2014).

39. Yang, X. *et al*. Oxygen vacancies induced special CO_2 adsorption modes on Bi_2MoO_6 for highly selective conversion to CH_4. *Appl. Catal. B: Environ.* **259**, 118088 (2019).

40. Yang, R., Fu, Y., Zhang, Y., Tsubaki, N. In situ DRIFT study of low-temperature methanol synthesis mechanism on Cu/ZnO catalysts from CO_2-containing syngas using ethanol promoter. *J. Catal.* **228**, 23-35 (2004).

41. Edwards, J. F., Schrader, G. L. Infrared spectroscopy of Cu/ZnO catalysts for the water-gas shift reaction and methanol synthesis. *J. Phys. Chem.* **88**, 5620-5624 (1984).
